# Immune gene signatures for predicting durable clinical benefit of anti-PD-1 immunotherapy in patients with non-small cell lung cancer

**DOI:** 10.1038/s41598-019-57218-9

**Published:** 2020-01-20

**Authors:** Sohyun Hwang, Ah-Young Kwon, Ju-Yeon Jeong, Sewha Kim, Haeyoun Kang, Joonsuk Park, Joo-Hang Kim, Ok Jin Han, Sun Min Lim, Hee Jung An

**Affiliations:** 1 Department of Pathology, CHA University, CHA Bundang Medical Center, Seongnam-si, Kyeonggi-do Republic of Korea; 2 CHA Advanced Research Institute, CHA Bundang Medical Center, Seongnam-si, Kyeonggi-do Republic of Korea; 3 Department of Thoracic Surgery, CHA University, CHA Bundang Medical Center, Seongnam-si, Kyeonggi-do Republic of Korea; 4 Hematology- Oncology, Department of Internal Medicine, CHA University, CHA Bundang Medical Center, Seongnam-si, Kyeonggi-do Republic of Korea

**Keywords:** Non-small-cell lung cancer, Tumour biomarkers

## Abstract

Immune checkpoint blockade is promising for treating non-small-cell lung cancer (NSCLC). We used multipanel markers to predict the response to immune checkpoint inhibitors (ICIs) by characterizing gene expression signatures or individual genes in patients who showed durable clinical benefit to ICIs. Twenty-one patients with NSCLC treated with single-agent anti-programmed cell death protein (PD)-1 antibody were analyzed and their clinicopathological characteristics and response to ICIs were characterized. Nine (43%) showed a durable clinical benefit (DCB), while the remaining 12 (57%) patients showed non-durable benefit (NDB). The M1 and peripheral T cell signatures showed the best performance for discriminating DCB from NDB (sensitivity, specificity, accuracy = 0.89, 1.0, 0.95, respectively). Progression-free survival (PFS) was significantly longer in patients with high M1 signature or high peripheral T cell signature scores. *CD137* and *PSMB9* mRNA expression was higher in the DCB group than in the NDB group. Patients with high PSMB9 expression showed longer PFS. M1 signature, peripheral T cell signature and high mRNA expression level of CD137 and PSMB9 showed better predictive performance than known biomarkers, such as PD-L1 immunohistochemistry, tumor mutation burden, or tumor-infiltrating lymphocytes.

## Introduction

Immune checkpoint inhibitors (ICIs) have improved the clinical outcomes of non-small-cell lung cancer (NSCLC) and have emerged as the most effective anticancer agents even in the first-line setting^1^. Immune checkpoint proteins, such as programmed death cell protein-1 (PD-1) or CTL associated antigen 4 (CTLA-4) can produce long-term durable remission in patients who respond to treatment^2–5^. Currently, the anti-PD-1 agent pembrolizumab is approved for use as first- and second-line therapy in patients with advanced NSCLC whose tumors express PD-L1 in immunohistochemical analysis^1,6^. Nivolumab (anti-PD-1) and atezolizumab (anti-PD-L1) are both indicated for use as second-line therapies regardless of PD-L1 expression^7,8^. However, antitumor efficacy is observed in 20–30% of patients with NSCLC, with most patients not achieving objective responses.

Tumor expression of PD-L1 has been most widely investigated as a predictive marker of response, but the sensitivity and specificity of this approach is modest^1,9^. While PD-L1 either on tumor or immune cells must be present for immune checkpoint therapy to be effective, PD-L1 testing shows variable results because of the different antibodies and cutoff values used^10^. PD-L1 alone cannot accurately reflect the complexity of the tumor microenvironment involved in the response to immunotherapy. Recent data have suggested that myeloid-derived suppressor cells, macrophages, and regulatory T cells are involved in determining the response to immune checkpoint inhibitors^11,12^.

At the genomic level, tumor mutation burden (TMB) has been correlated with the clinical response to anti-PD-1 therapy and associated with favorable responses in smokers^13^. However, TMB alone does not directly lead to neoepitope processing by major histocompatibility complex (MHC) class molecules and the range of neoepitope loads of responders overlaps significantly with those of non-responders. Additionally, the optimal cut-off of mutation load is controversial, with different cutoff values used in different clinical trials and research-based assays^14,15^.

Previously established biomarkers for predicting clinical outcomes of immunotherapy, such as PD-L1 expression, have not guaranteed success for all patients, and thus further studies are needed to identify the most accurate and predictive signatures in each patient^16^. Previous studies demonstrated that immunogenic gene expression is correlated with the response to therapy^17,18^. In this study, we analyzed the transcriptomes of tumor tissues before treatment by using an immune profile panel to identify factors that may influence sensitivity or resistance to ICIs.

## Results

### Immune landscape of NSCLC tumors

To investigate immune-related gene expression signatures associated with the response to ICIs, we prospectively collected pretreatment tumor samples from patients with metastatic NSCLC. All patients had been treated with PD-1 inhibitors and their clinicopathological characteristics are summarized in Table 1. The cohort included squamous cell carcinoma (n = 8), adenocarcinoma (n = 9), large cell neuroendocrine carcinoma (n = 3), and adenosquamous carcinoma (n = 1) by histology; 1 tumor harbored an *EGFR* activating mutation. Sixteen (77%) patients had a current or former smoking history. PD-L1 expression according to IHC revealed values of 0% in 6 (28%) patients, ≤1– <50% in 9 (43%) patients, and ≥50% in 6 (28%) patients. Of the 21 patients, 9 (43%) achieved a durable clinical benefit, as per RECIST v1.1, and the remaining 12 (57%) patients showed no durable benefit. One patient achieved a complete response (CR) on ICI and is being administered therapy (PFS for 32 + months). The median PFS of all patients was 2.2 months (95% CI, 1.4–3.0), while the median PFS of DCB and NDB was 11.2 months (95% CI, 6.4–16.1), and 1.6 months (95% CI, 0.7–2.5), respectively. The median OS of all patients was 33.1 months (95% CI, 9.4–56.8), while the median OS of DCB and NDB was 41.8 months (95% CI, 33.5–50.2) and 13.7 months (95% CI, 5.4–22.0), respectively.

**Table 1:** Baseline clinical characteristics.

Patient characteristics (N = 21)	DCB (N = 9)	NDB (N = 12)
Age (years), median (range)	64 (58–79)	64 (46–71)
**Gender**		
Male	7 (78%)	8 (67%)
Female	2 (22%)	4 (33%)
**Histology**		
Non-squamous	7 (78%)	5 (42%)
Squamous	2 (22%)	7 (58%)
**Smoking status**		
Current/former	7 (78%)	9 (75%)
Never	2 (22%)	3 (25%)
**Stage**		
IIIB	3 (33%)	4 (33%)
IV	6 (67%)	8 (67%)
**PD-L1 expression**		
0%	2 (22%)	4 (33%)
1–50%	4 (44%)	5 (42%)
≥50%	3 (33%)	3 (25%)
**Genotypes**		
EGFR mutation	0	1 (1%)
ALK fusion	0	0
**Prior systemic therapy**		
1	5 (50%)	5 (46%)
2	2 (20%)	2 (18%)
3	1 (10%)	2 (18%)
≥4	1 (10%)	2 (18%)

**Figure 1:**
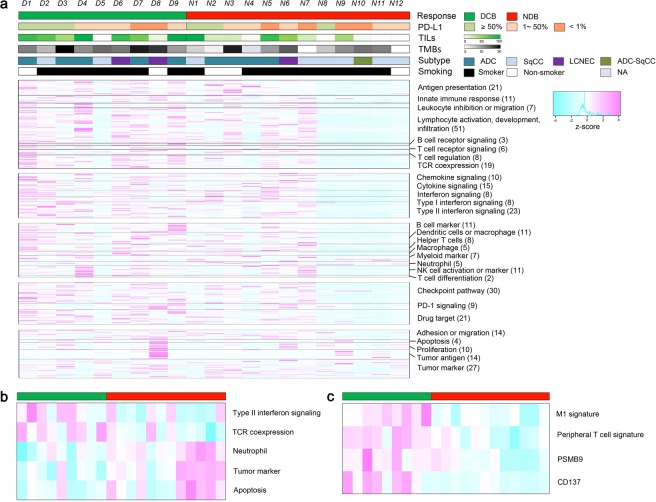
Heatmap for the 395 immune-related genes from 21 patients. (**a**) Columns represent patients and rows genes. Expression levels have been aligned according to functional annotation groups. Categories of response (DCB, NDB), PD-L1 expression (≥50%,1–50%, <1%), smoking status (smoker, non-smoker) and histologic subtypes (adenocarcinoma, squamous cell carcinoma, large cell neuroendocrine carcinoma, adenosquamous cell carcinoma) are shown, and tumor infiltrating lymphocytes (TILs) and tumor mutation burden (TMB) are shown in continuous variables. (**b)** Heatmap for five signature scores of functional annotation groups discriminating DCB and NDB patients. (**c)** Heatmap for two gene signature scores and two genes that discriminate DCB and NDB patients.

We explored the immune landscape for predicting the response to ICI in these samples using a panel of 395 immune-related genes with the Oncomine Immune Response Research Assay (Fig. 1). Among the 395 genes, 2 genes which were not expressed in any patients, and 11 housekeeping genes were excluded from the plot. We aligned the results of 382 genes according to functional annotation groups such as lymphocyte regulation, cytokine signaling, lymphocyte markers, checkpoint pathway, tumor characterization, and housekeeping. In Fig. 1a, individual patients are represented in each column, organized by DCB on the left (green) and NDB on the right (red). We observed high expression of genes in DCB cases, suggesting pre-existing immune recognition of the tumor. Notably, 7 patients (N1–N7) in the NDB group also had relatively higher expression of genes related to lymphocyte regulation, cytokine signaling, lymphocyte markers, and checkpoint pathway (named as ‘hot NDB’ in contrast to ‘cold NDB’).

We also calculated the gene signature score of genes in each functional annotation in Fig. 1a and compared the differences between DCB and NDB patients in Fig. 1b. Type II interferon signaling and T cell receptor co-expression were highly expressed in DCB patients, whereas neutrophil, tumor marker, and apoptosis were highly expressed in NDB patients. The predictability of each gene signature is shown in Supplementary Table S1.

Previously identified predictive biomarkers of response such as PD-L1 expression, TMB, and TILs were also compared between the two groups. The median TMB did not significantly differ between the DCB and NDB groups: 11.76 *vs*. 8.4 mutations per Mb, respectively (Mann-Whitney *P* = 0.08). TMB was also compared according to PD-L1 expression and showed median values of 10.94, 10.93, and 6.29/Mb, respectively for PD-L1 expression of <1%, 1–50%, ≥ 50% (Supplementary Fig. S1A,B**)**. The proportions of DCB were 33%, 44%, and 50% for PD-L1 expression of <1%, 1–50%, and ≥50%; there was no significant difference between the three groups (Supplementary Fig. S1C). In agreement with a previous report^13^, patients with a higher TMB showed a prolonged PFS compared to patients with a lower TMB (16.78 months *vs*. 7.14 months, *P* = 0.003) (Supplementary Fig. S2). Although not a previously identified biomarker, we analyzed the proportion of core TILs among three groups: DCB, hot NDB, and cold NDB. This value was significantly higher in the DCB group than in the NDB group (*P* = 0.03). Comparison of PD-L1 expression and TILs revealed no significant difference among the three groups. While the DCB and “hot” NDB groups showed no significant difference, the “cold” NDB group showed significantly lower core TILs than DCB (*P* = 0.02) and “hot” NDB (*P* = 0.04) (Supplementary Fig. S3A–C). The ratio of core TIL to total TIL was significantly different between the DCB and NDB groups (*P* = 0.03), the “hot” NDB group showed no difference from the DCB group, and the “cold” NDB group showed a lower core TIL ratio than the DCB (*P* = 0.002) and “hot” NDB groups (*P* = 0.03) (Supplementary Fig. S3D**)**.

### Major gene signatures associated with therapeutic response to anti-PD-1 and patient prognosis

**Figure 2:**
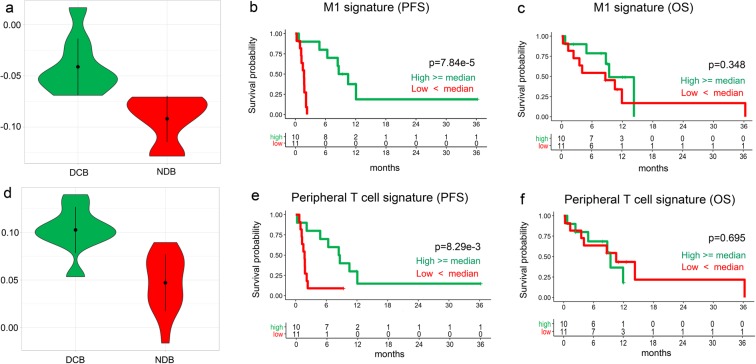
RNA expressions of gene signatures associated with therapeutic response and improved outcome to anti-PD-1. (**a)** The expression of M1 signature is significantly higher in DCB group compared to NDB group (t-test, *P* = 4.95e^−4^). (**b)** Patients with high M1 signature have significantly prolonged progression-free survival than those with low M1 signature (log-rank, *P* = 7.84 e^−4^). (**c)** Patients with high M1 signature have similar overall survival than those with low M1 signature (log-rank, *P* = 0.348). (**d)** The expression of peripheral T cell signature was significantly higher in DCB group compared to NDB group (t-test, *P* = 1.08e^−4^). (**e)** High peripheral T cell signature was associated with significantly prolonged progression-free survival (log-rank, *P* = 8.29e^−3^). (**f)** Patients with high peripheral T cell signature have similar overall survival than those with low signature (log-rank, *P* = 0.695).

Next, pre-existing gene signatures of immune response were evaluated (See Methods). From the results of random forest analysis of the gene signature data, we identified two major gene signatures (GS) showing marked expression differences depending on the response to ICI and named each signature as the M1 signature and peripheral T cell signature, respectively (Fig. 1c, Supplementary Table S2). The M1 signature genes included *CBLB*, *CCR7*, *CD27*, *CD48*, *FOXO1*, *FYB*, *HLA-B*, *HLA-G*, *IFIH1*, *IKZF4*, *LAMP3*, *NFKBIA*, and *SAMHD1*, while the peripheral T cell signature included *HLA-DOA*, *GPR18*, and *STAT1*. These two gene signatures showed the best performance for discriminating DCB from NDB (Supplementary Fig. S4A, sensitivity = 0.89, specificity = 1.0, and accuracy = 0.95). Only one patient in DCB was misclassified as NDB according to these two signatures. The M1 signature (GSE5099 M1 vs. M2) and peripheral T cell signature (GSE7852 LN vs. FAT Tconv up) were significantly increased in the DCB group compared to in the NDB group (*P* = 4.95e-4 and 1.08e-4, respectively by *t*-test analysis; Fig. 2a,d). To investigate the effect of two GSs on patient survival, patients were dichotomized into high or low score groups based on the respective median GS scores. Kaplan-Meier plots indicated that PFS was significantly longer in patients with high M1 signature scores, although OS was not different (Fig. 2b,c). Similarly, PFS was significantly longer in patients with high peripheral T cell signatures (Fig. 2e), although OS was not different (Fig. 2f).

### Genes associated with therapeutic response to anti-PD-1 and patient prognosis

**Figure 3:**
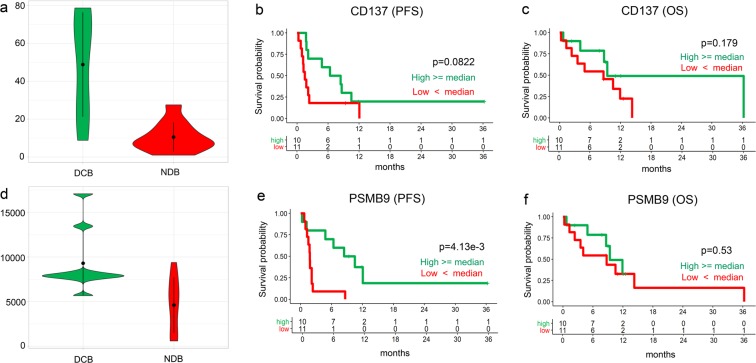
RNA expressions of the genes associated with therapeutic response and improved outcome to anti-PD-1. **(a)** The RNA expression of the gene CD137 is significantly higher in DCB group compared to NDB group (t-test, *P* = 2.78e^−3^) (**b)**. The Kaplan-Meier plots of the patients with high and low RNA expression of each gene indicated that the PFS is higher in the patients with high expressions of CD137, but not statistically significant (log-rank, *P* = 0.082) (**c)**. The OS does not show statistically significant difference for CD137 (log-rank *P* = 0.179). (**d)** The RNA expression of the PSBM9 gene is significantly higher in DCB group compared to NDB group (t-test, P = 6.66e^−3^).(**e)**. PFS is significantly higher in patients with high PSMB9 RNA expression (log-rank, P = 4.13e^−3^) (**f)**. OS is similar between two groups (log-rank *P* = 0.53).

We analyzed RNA expression data to identify significant genes that may help predict the response to ICI in patients with lung cancer. When we constructed random forest models by increasing the gene numbers from two genes to all 393 expressed genes in our samples and measured its prediction performance by leave-one-out cross-validation, the model of three genes (*CD137*, *PSMB9*, and *BCL-2*) showed the best performance (Supplementary Fig. S4B, sensitivity = 0.67, specificity = 0.75, and accuracy = 0.71). Among these 3 genes, the expression of 2 genes, *CD137* and *PSMB9*, was higher in the DCB group than in the NDB group (Figs. 1c and 3a,d). The survival of patients with high and low (higher and lower than the median) RNA expression of CD137 and PSMB9 was further analyzed. Kaplan-Meier plots indicated that the median PFS and OS were similar among patients with high and low CD137 mRNA expression (Fig. 3b,c). Of note, PFS was longer in patients with high PSMB9 RNA expression (Fig. 3e), which suggests that high RNA expression of PSMB9 may predict patients who will achieve a durable clinical response to ICI.

### Comparison of predictive abilities of PD-L1 expression, TILs, TMB, genes, and gene signatures

**Figure 4:**
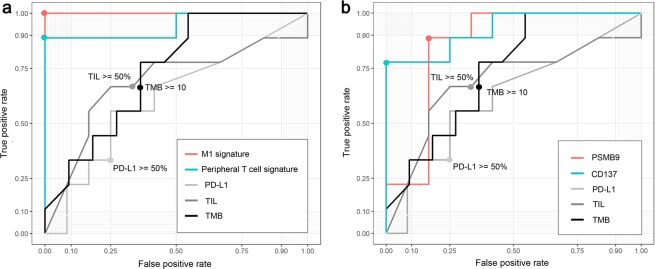
Assessment of each marker as predictive biomarker of durable clinical benefit to anti-PD-1 therapy. (**a**) Predictive abilities of M1 signature, peripheral T cell signature, PD-L1 expression, tumor infiltrating lymphocytes (TIL), and tumor mutation burden (TMB) are compared by receiver operating characteristic (ROC) curves and area under the curve (AUC) values. The points on the curves of PD-L1 expression, TIL, and TMB indicates the cutoff values widely used. (**b)** Predictive abilities of PSMB9, CD137, PD-L1 expression, TIL, and TMB were compared by ROC curves and AUC values.

PD-L1 IHC staining and TILs are established markers for predicting the response to ICI therapy. We investigated the predictive abilities of our new methods of two gene signatures and two genes. We investigated whether the response to ICI therapy could be predicted and compared the predictive abilities of each method by ROC curves and AUC values. For PD-L1 expression and TILs, the AUC values were near 0.6 (AUC values = 0.62 and 0.69, respectively). The AUC value of TMB was 0.74, which was slightly higher than those of PD-L1 expression and TILs. We compared the ROC curves of two gene signatures and two genes with those of the three known methods. For the two gene signatures (Fig. 4a) and two genes (Fig. 4b), higher AUC values were observed than for conventional methods such as PD-L1 expression, TILs, and TMB: M1 signature, AUC values were 1; peripheral T cell signature, 0.94; CD137, 0.93; and PSMB9, 0.85. The predictability of each gene signature and the selected genes determined by *t*-test, edgeR, AUC, survival analysis is summarized in Supplementary Table S3.

## Discussion

In this study, we found that multigene immune signatures including the peripheral T cell signature and M1 signature were enriched in the DCB group and can define the tumor microenvironment that predicts a clinical benefit to anti-PD-1 therapy. In addition to the two gene signatures, *CD137* and *PSBM9* were independently predictive of clinical benefits. This is the first study to report the predictability of selected gene signatures and genes for discriminating DCB from NDB, indicating that integrated multigene signatures are better predictors than PD-L1 status or TMB per Mb information.

The spectrums of genes associated with the two signatures suggest a complex immune response in anti-PD-1 responsive tumors. The peripheral T cell signature comprised of HLA-DOA, GPR18, and STAT1 indicated that the activated T cell and its downstream signaling molecule, STAT1, plays a key role in antitumor responses. HLA-DOA corresponding to MHC class II specifically presents antigens to T-helper cells (CD4^+^ T cells), and recent data suggested the importance of MHC class II in antitumor activity^19,20^, as CD4^+^ T cells can kill tumors both by directly binding to MHC II-expressing tumor cells and indirectly by activating tumor-infiltrating macrophages.

Tumor-associated macrophages play a central role in tumor progression and metastasis and their plasticity enables their classification along a M1-M2 polarization axis^21^. Our M1 signature highlights the importance of M1 polarization by including CD48, which is utilized by M1 macrophages to trigger natural killer (NK) cell production of interferon (IFN)-γ. IFN-γ can upregulate HLA molecules and antigen-presenting machinery such as PSMB9 (LMP2). PSBM9 constitutes the ß-subunits of the proteasome, which generates MHC-restricted peptides^22^. CD137 (4–1BB, TNFRSF9) is expressed on activated T cells and NK cells and is a potent co-stimulator of antitumor immune responses^23^. CD137-CD137L signaling is the main driver of cellular immunity by enhancing T and NK cell activity, and clinical trials of CD137 agonists are currently underway to assess their efficacy either as single agents or in combination with ICIs or vaccines. The association of PSMB9 and CD137 with the clinical response suggests that additional aspects of antigen presentation and NK cell biology are involved in determining the immune response.

When we compared our results with other ICI-treated, non-NSCLC cohort to validate our study, we found the mRNA data of 51 pre-ICI treated melanoma patients and its clinical outcome by Riaz *et al*.^24^, which was in accordance with our results for two genes and of two signatures. They demonstrated that expression of CD137 in response group (complete or partial response) was higher than that in non-responsive group (progressive or stable disease), but it was not statistically significant (p-value: 0.11). The results of PSMB9, M1 signature and peripheral T cell signature were also similar to ours, showing higher expression in responsive group, but not statistically significant.

Furthermore, our data indicate that the NDB group can be subdivided to 2 groups: 5 patients had “cold” TME, devoid of an immune response and signaling molecules, and 7 patients had variable degrees of “hot” TME. The reason for the lack of response in this subset of inflamed NDB is unclear, as 4 (57%) of these patients presented with heavy and rapid progression of tumor burden prior to ICI therapy. In our study, the proportion of core TILs could distinguish the cold NDB group from the DCB and hot NDB groups. However, there were no significant differences between DCB and hot NDB in terms of core TILs. We assumed that the effector T cells in hot NDB were already exhausted; for example, these cells lacked the signaling molecules necessary to function or that the tumor cells had more powerful defense mechanisms to inhibit the action of immune cells, providing immunosuppressive environments to promote immune evasion. Further studies are necessary to explain the lack of response in hot NDB patients.

Other genomic mechanisms determining responses such as defects in IFN signaling through *JAK1/2*-inactivating mutations or defective HLA class antigen processing through deleterious mutations in Beta-2 microglobulin may be involved^25^. As recently suggested by Richard *et al*., further exome-based analysis may identify parameters for more accurately predicting outcomes to ICI therapy^26^. Similarly, some immune evasion mechanisms such as transforming growth factor ß signaling^27^ or indoleamine 2,3-dioxygenase activity may influence the ICI response^28^.

Although PD-L1 and TMB are among the most validated biomarkers to ICI response^29,30^, the antitumor immune response is complex and may not be fully captured by a single biomarker. Therefore, multiplex approaches including inflammatory gene signatures may more accurately represent immunophenotypic features indicative of ICI benefits. While TMB may contribute to the neoantigenic load, it does not directly represent neoantigens that can be recognized by the immune system as ‘neoepitopes’ presented on MHC molecules. Similarly, both PD-L1 and TMB were inferior for predicting durable clinical benefits in our study population.

Although some previous studies^31^ demonstrated show different immune environment according to different histologic subtypes, we analyzed both types of squamous cell carcinoma and adenocarcinoma together in the present study because several large-scale, clinical trials, such as the open-label, randomized, phase III CheckMate017 trial showed the similar objective response rates to immune checkpoint inhibitors in both subtypes. Moreover, since our primary objective was to find predictive biomarkers that determine durable response to immune checkpoint inhibitors, we think that pooled analysis of both squamous and nonsquamous histologies is feasible, especially in this small-scaled study.

Some limitations of our study were the heterogeneity of tumor sites analyzed and variable time points of tissue acquisition. Intervening treatments were administered to 14% of patients after biopsy was taken, and radiotherapy and chemotherapy may upregulate tumor PD-L1 expression or cause a more inflammatory tumor microenvironment^32,33^. Although the optimal time window or tumor site that best represents the tumor microenvironment is unknown, future studies analyzing prospectively collected tissue samples with large patient numbers are required to validate our results. Another limitation of this study was that our multiplex panel contains selected key genes expressed in the tumor microenvironment, and thus may only capture limited data compared to whole transcriptome sequencing.

Immune reaction to tumor is a complicated and sophisticated process that involves numerous factors and pathways, organically affecting each other. Multiple features are involved in the response of ICIs, such as antigens in tumor cells, amounts of immune cells, antigen-recognition, and the interaction between tumor and immune cells. For ICIs to work in tumor, we need generation of tumor-reactive T cells, activation of effector T cell function, and formation of effector memory T cells.^34^ Although we found two multigene immune signatures and two genes for predictive markers of the response to ICI, those may not explain all. Of note, we identified significant differences in the two gene signatures and two genes between DCB and NDB groups which may provide a clue to understanding the interaction between tumor and host’s anti-tumor immune system.

In conclusion, our study identified composite predictive biomarkers, M1 and peripheral T cell signatures, for identifying patients who will have a durable clinical benefit with good sensitivity and specificity, and future validation studies are necessary to determine their value in a prospective cohort.

## Methods

### Case and patient samples

Cases showing a partial or complete response to anti-PD-1 antibody (nivolumab or pembrolizumab) by Response Evaluation Criteria in Solid Tumor (RECIST) v1.1 lasting >24 weeks or stable disease lasting >24 weeks were considered as having a durable clinical benefit (DCB). Cases showing progression of disease or stable disease lasting ≤24 weeks were considered as showing no durable benefit (NDB). Progression-free survival (PFS) is defined as length of time from the start of the anti-PD-1 antibody until progression. Overall survival (OS) is defined as the length of time from the date of diagnosis until death.

A total of 34 patients with NSCLC who were treated with a single-agent anti-PD-1 antibody were initially recruited. Two board-certified pathologists (H.K and S.K.) reviewed the hematoxylin and eosin (H&E)–stained slides of all patients to evaluate tissue quality and identify the tumor area to be examined. Eight specimens were excluded because of inadequate tumor cells for sequencing. Twenty-six specimens were applied to gene extraction, however, five specimens showed poor sequencing quality and were excluded. Finally, twenty-one specimens (9 DCB and 12 NDB) with adequate tumor tissues were finally selected for sequencing, which included 10 needle biopsies and 11 surgical resections. This study was approved by the institutional review board of CHA Bundang Medical Center (IRB file No. CHAMC 2018-02-032), and all experiments were performed in accordance with the guidelines and regulations of IRB of CHA Bundang Medical Center. Informed consent was obtained from each patient prior to surgery.

### RNA and DNA extraction

For RNA and DNA extraction, 2–10 sections of 10 µm-thick formalin-fixed paraffin-embedded tissue were prepared. After macrodissection of the tumor area, RNA and DNA were extracted using the Recover All Total Nucleic Acid Isolation kit (Thermo Fisher Scientific, Waltham, MA, USA) according to the manufacturer’s protocol. RNA concentrations were determined with the Qubit RNA HS Assay Kit and Qubit 3.0 Fluorometer (Thermo Fisher Scientific). DNA concentrations were measured with the Qubit dsDNA HS Assay Kit and Qubit 3.0 Fluorometer (Thermo Fisher Scientific).

### Library preparation and RNA sequencing

RNA was reverse-transcribed into cDNA using the SuperScript VILO™ cDNA synthesis kit (Thermo Fisher Scientific). Libraries were prepared manually using the Ion AmpliSeq™ Library kit 2.0 (Life Technologies, Carlsbad, CA, USA) and Oncomine™ Immune Response Research Assay (Thermo Fisher Scientific). A 50-pM pool of RNA libraries was used for sequencing of a 395-gene panel focused on diverse immunological processes including tumor infiltration by immune cells, and other key immune functions (Supplementary Table S4). Template preparation and enrichment were performed using an Ion Chef™ system (Thermo Fisher Scientific) and Ion 520™ & Ion 530™ Kit – Chef (Thermo Fisher Scientific). Sequencing was performed on an Ion S5™ XL Sequencer using an Ion 530 Chip and Ion S5™ sequencing kit (all from Thermo Fisher Scientific). Alignment of the sequences to the reference immuneresponse_V3.1 and counting of the sequencing reads were performed using the ImmuneResponseRNA Report plug-in in Torrent Suite software (Version 5.2).

### PD-L1 immunohistochemical staining assay

Immunohistochemical (IHC) staining for PD-L1 (SP263) was performed on a Ventana Benchmark automated staining platform (Ventana Medical Systems, Inc., Tucson, AZ, USA) using a VENTANA OptiView diaminobenzidine tetrahydrochloride IHC Detection Kit (P/N 760–700) and its staining protocol. The formalin-fixed paraffin-embedded tissues of all cases were cut, dried, deparaffinized, rehydrated, and heated following the protocol. Negative control slides were also stained using a matched rabbit immunoglobulin G. Normal-term placenta tissue was included as a positive staining control on each slide. IHC staining was interpreted by two independent, board-certified pathologists, blinded to the clinical data and patient outcomes (A.Y.K. and S.K.). Any discordance between the two pathologists did not observed.

### Evaluation of tumor-infiltrating lymphocytes

Histologic evaluation of tumor-infiltrating lymphocytes (TILs) was performed on H&E-stained slides. Three pathologists (A.Y.K., H.K., and S.K.) who were blinded to the clinical data and patient outcomes assessed the TILs. The percentage of tumor and tumoral stroma containing mononuclear immune cells was evaluated according to recommendations previously proposed by an International TIL Working Group^35^. Additionally, core TILs and marginal TILs were estimated together: core TILs were highly infiltrated immune cells within the tumor core and in contact with tumor cells, which was described as infiltrated-inflamed cells, and marginal TILs presented along the margin of tumor-cell clusters or in fibrotic nests, which were described as infiltrated-excluded cells^36^. A representative image is shown in Supplementary Fig. S5.

### Tumor mutation burden

The Oncomine™ Tumor Mutation Load Assay is a PCR-based target enrichment next-generation sequencing assay performed on the Ion Torrent platform. The panel covers 1.65 Mb with 1.2 Mb of exonic bases across 409 oncogenes relevant across major cancer types. DNA was extracted from formalin-fixed paraffin-embedded tissue using the RecoverAll Multi-Sample RNA/DNA Workflow kit (Invitrogen, Carlsbad, CA, USA), following the manufacturer’s protocol. Genomic DNA was quantified by quantitative real-time reverse transcription (RT)-PCR using a TaqMan® RNase P Detection Reagents kit (Applied Biosystems, Foster City, CA, USA). The Libraries were prepared by automation using The Ion AmpliSeq™ Kit for Chef DL8 (Ion Torrent, Thermo Fisher Scientific). Library preparation and templating were performed on the Ion Chef using an automated Ion Ampliseq^TM^ Kit for Chef DL8 (Ion Torrent) and Ion 540 chip - Chef kit (Ion Torrent) respectively. Sequencing was performed on the Ion S5™XL Sequencer using an Ion S5™sequencing kit (Thermo Fisher Scientific) with Torrent Suite software (Version 5.10). Variants were identified using the Ion Torrent Variant Caller plug-in (Version 5.10) and annotated using Ion Reporter software (Version 5.10).

### RNA sequencing data analysis

Of the twenty-six samples included in sequencing, we removed five samples for which total read counts were less than 1 million and combined absolute read count data for each sequencing data run. We calculated the TPM (Transcripts Per Million) by normalizing each gene length and the total read counts^37^, and then measured the gene signature score of genes in each functional annotation in Fig. 1a and Supplementary Table S1 by ssGSEA in GSVA R package^38^. For the predictability of each gene signature, the area under the receiver operating characteristic (ROC) curve (AUC), survival and two sample *t*-test analyses were performed. For multiple comparisons, we used the Bejamini-Hochberg procedure^39^.

### Random forest and gene signature analysis

Gene expression data were normalized by TPM measure and random forest analysis was performed with the randomForest^40^ and caret R packages. The number of trees was 10,000 and we used default values for other parameters. For performance measurement, leave-one-out cross-validation (LOOCV) method was conducted. We also conducted random forest analysis for the gene signature data. Gene signature sets consisted of five data sets; Gene Ontology Biological Process, downloaded on June, 21, 2018^41^, immune landscape gene sets^42^, immunoscore of each immune cell^43^, hallmark of cancer^44^, and immunologic signature of mSigDB 6.1^45^. Gene signature scores were calculated by ssGSEA in GSVA R package^38^.

### The predictive abilities of candidate genes and gene signatures

The predictability of two genes (*CD137* and *PSMB9*) and of two gene signatures (M1 signature and peripheral T cell signature) were determined by *t*-test, edgeR^46^, AUC and survival analyses. For edgeR analysis, we normalized raw read counts according to edgeR quasi-likelihood pipeline and for other analyses; we used gene expression data normalized by TPM measure.

### Statistical analysis

Heatmap analysis was carried out with gplots R package. All plots such as violin plots and survival plots were depicted in ggplot2 R package^47^. Survival analysis was conducted using the survival^48^ and survminer R packages and the *P*-value of each Kaplan Meier-plot was calculated by log-rank test. AUC was calculated with the ROCR^49^ and plotROC R packages^50^. All statistical data were analyzed using R 3.4.4.

### Accession codes

All expression data available at GEO Database (https://www.ncbi.nlm.nih.gov/geo/) with accession number GSE136961.

## Supplementary information

Supplementary Information.

## References

[1] Reck M (2016). Pembrolizumab versus Chemotherapy for PD-L1-Positive Non-Small-Cell Lung Cancer. N. Engl. J. Med..

[2] Gettinger SN (2015). Overall Survival and Long-Term Safety of Nivolumab (Anti-Programmed Death 1 Antibody, BMS-936558, ONO-4538) in Patients With Previously Treated Advanced Non-Small-Cell Lung Cancer. J. Clin. Oncol..

[3] Brahmer JR (2012). Safety and activity of anti-PD-L1 antibody in patients with advanced cancer. N. Engl. J. Med..

[4] Hodi FS (2010). Improved survival with ipilimumab in patients with metastatic melanoma. N. Engl. J. Med..

[5] McDermott DF (2015). Survival, Durable Response, and Long-Term Safety in Patients With Previously Treated Advanced Renal Cell Carcinoma Receiving Nivolumab. J. Clin. Oncol..

[6] Herbst RS (2016). Pembrolizumab versus docetaxel for previously treated, PD-L1-positive, advanced non-small-cell lung cancer (KEYNOTE-010): a randomised controlled trial. Lancet.

[7] Borghaei H (2015). Nivolumab versus Docetaxel in Advanced Nonsquamous Non-Small-Cell Lung Cancer. N. Engl. J. Med..

[8] Rittmeyer A (2017). Atezolizumab versus docetaxel in patients with previously treated non-small-cell lung cancer (OAK): a phase 3, open-label, multicentre randomised controlled trial. Lancet.

[9] Taube JM (2014). Association of PD-1, PD-1 ligands, and other features of the tumor immune microenvironment with response to anti-PD-1 therapy. Clin. Cancer Res..

[10] Mehnert JM (2017). The Challenge for Development of Valuable Immuno-oncology Biomarkers. Clin. Cancer Res..

[11] Weber R (2018). Myeloid-Derived Suppressor Cells Hinder the Anti-Cancer Activity of Immune Checkpoint Inhibitors. Front. Immunol..

[12] Darvin P, Toor SM, Sasidharan Nair V, Elkord E (2018). Immune checkpoint inhibitors: recent progress and potential biomarkers. Exp. Mol. Med..

[13] Rizvi NA (2015). Cancer immunology. Mutational landscape determines sensitivity to PD-1 blockade in non-small cell lung cancer. Science.

[14] Hellmann MD (2018). Nivolumab plus Ipilimumab in Lung Cancer with a High Tumor Mutational Burden. N. Engl. J. Med..

[15] Hellmann MD (2018). Genomic Features of Response to Combination Immunotherapy in Patients with Advanced Non-Small-Cell Lung Cancer. Cancer Cell.

[16] Gnjatic S (2017). Identifying baseline immune-related biomarkers to predict clinical outcome of immunotherapy. J. Immunother. Cancer.

[17] Bindea G (2013). Spatiotemporal dynamics of intratumoral immune cells reveal the immune landscape in human cancer. Immunity.

[18] Ayers M (2017). IFN-gamma-related mRNA profile predicts clinical response to PD-1 blockade. J. Clin. Invest..

[19] Kreiter S (2015). Mutant MHC class II epitopes drive therapeutic immune responses to cancer. Nature.

[20] Ott PA (2017). An immunogenic personal neoantigen vaccine for patients with melanoma. Nature.

[21] Genard G, Lucas S, Michiels C (2017). Reprogramming of Tumor-Associated Macrophages with Anticancer Therapies: Radiotherapy versus Chemo- and Immunotherapies. Front. Immunol..

[22] Gaczynska M, Rock KL, Spies T, Goldberg AL (1994). Peptidase activities of proteasomes are differentially regulated by the major histocompatibility complex-encoded genes for LMP2 and LMP7. Proc. Natl Acad. Sci. USA.

[23] Dharmadhikari B (2016). CD137 and CD137L signals are main drivers of type 1, cell-mediated immune responses. Oncoimmunology.

[24] Riaz N (2017). Tumor and Microenvironment Evolution during Immunotherapy with Nivolumab. Cell.

[25] Zaretsky JM (2016). Mutations Associated with Acquired Resistance to PD-1 Blockade in Melanoma. N. Engl. J. Med..

[26] Richard C (2019). Exome Analysis Reveals Genomic Markers Associated with Better Efficacy of Nivolumab in Lung Cancer Patients. Clin. Cancer Res..

[27] Mariathasan S (2018). TGFbeta attenuates tumour response to PD-L1 blockade by contributing to exclusion of T cells. Nature.

[28] Banerjee T (2008). A key *in vivo* antitumor mechanism of action of natural product-based brassinins is inhibition of indoleamine 2,3-dioxygenase. Oncogene.

[29] Goodman AM (2017). Tumor Mutational Burden as an Independent Predictor of Response to Immunotherapy in Diverse Cancers. Mol. Cancer Ther..

[30] Kluger HM (2017). PD-L1 Studies Across Tumor Types, Its Differential Expression and Predictive Value in Patients Treated with Immune Checkpoint Inhibitors. Clin. Cancer Res..

[31] Seo JS, Kim A, Shin JY, Kim YT (2018). Comprehensive analysis of the tumor immune micro-environment in non-small cell lung cancer for efficacy of checkpoint inhibitor. Sci. Rep..

[32] Kordbacheh T, Honeychurch J, Blackhall F, Faivre-Finn C, Illidge T (2018). Radiotherapy and anti-PD-1/PD-L1 combinations in lung cancer: building better translational research platforms. Ann. Oncol..

[33] Peng J (2015). Chemotherapy Induces Programmed Cell Death-Ligand 1 Overexpression via the Nuclear Factor-kappaB to Foster an Immunosuppressive Tumor Microenvironment in Ovarian Cancer. Cancer Res..

[34] Jenkins RW, Barbie DA, Flaherty KT (2018). Mechanisms of resistance to immune checkpoint inhibitors. Br. J. Cancer.

[35] Salgado R (2015). The evaluation of tumor-infiltrating lymphocytes (TILs) in breast cancer: recommendations by an International TILs Working Group 2014. Ann. Oncol..

[36] Binnewies M (2018). Understanding the tumor immune microenvironment (TIME) for effective therapy. Nat. Med..

[37] Wagner GP, Kin K, Lynch VJ (2012). Measurement of mRNA abundance using RNA-seq data: RPKM measure is inconsistent among samples. Theory Biosci..

[38] Hanzelmann S, Castelo R, Guinney J (2013). GSVA: gene set variation analysis for microarray and RNA-seq data. BMC Bioinforma..

[39] Benjamini Y, Drai D, Elmer G, Kafkafi N, Golani I (2001). Controlling the false discovery rate in behavior genetics research. Behav. Brain Res..

[40] Wiener, A. L. A. M. *Classification and Regression by randomForest*. Vol. 2 18–22 (R News 2002).

[41] Ashburner M (2000). Gene ontology: tool for the unification of biology. The Gene Ontology Consortium. Nat. Genet..

[42] Thorsson V (2018). The Immune Landscape of Cancer. Immunity.

[43] Rooney MS, Shukla SA, Wu CJ, Getz G, Hacohen N (2015). Molecular and genetic properties of tumors associated with local immune cytolytic activity. Cell.

[44] Liberzon A (2015). The Molecular Signatures Database (MSigDB) hallmark gene set collection. Cell Syst..

[45] Liberzon, A. *Molecular signature database (MSigDB) 3.0*. Vol. 27 (Bioinformatics, 2011). 10.1093/bioinformatics/btr260 PMC3106198 21546393

[46] McCarthy DJ, Chen Y, Smyth GK (2012). Differential expression analysis of multifactor RNA-Seq experiments with respect to biological variation. Nucleic Acids Res..

[47] Wickham, H. *Elegant Graphics for Data Analysis*. (Springer-Verlag 2016).

[48] Terry, M. & Therneau, P. M. G. *Modeling Survival**Data: Extending the Cox Model*. (Springer, 2000).

[49] Sing, T. S. O., Beerenwinkel, N. & Lengauer, T. *ROCR: visualizing classifier performance in R*. Vol. 20 7881 (Bioinformatics, 2005). 10.1093/bioinformatics/bti623 16096348

[50] Sachs, M. C. *plotROC: A Tool for Plotting ROC Curves. Journal of Statistical Software*. Vol. 79 1–19 (Code Snippets, 2017). 10.18637/jss.v079.c02 PMC6347406 30686944

