# Supplementary material for: Immune gene signatures for predicting durable clinical benefit of anti-PD-1 immunotherapy in patients with non-small cell lung cancer
Source: Sci Rep. 2020 Jan 20;10:643. doi: 10.1038/s41598-019-57218-9 (PMC6971301; doi:10.1038/s41598-019-57218-9)
Supplement: Supplementary file 1 — Supplementary Information. [file 41598_2019_57218_MOESM1_ESM.docx]

**Immune gene signatures for predicting durable clinical benefit of anti-PD-1 immunotherapy in patients with non-small cell lung cancer**

Sohyun Hwang^1*^, Ah-Young Kwon^1*^, Ju-Yeon Jeong^2^, Sewha Kim^1^, Haeyoun Kang^1^, Joonsuk Park^3^, Joo-Hang Kim^4^, Ok Jin Han^2^, Sun Min Lim^4§^, Hee Jung An^1§^

^1^Department of Pathology, CHA University, CHA Bundang Medical Center, Seongnam-si, Kyeonggi-do, Republic of Korea

^2^CHA Advanced Research Institute, CHA Bundang Medical Center, Seongnam-si, Kyeonggi-do, Republic of Korea

^3^Department of Thoracic Surgery, CHA University, CHA Bundang Medical Center, Seongnam-si, Kyeonggi-do, Republic of Korea

^4^Hematology- Oncology, Department of Internal Medicine, CHA University, CHA Bundang Medical Center, Seongnam-si, Kyeonggi-do, Republic of Korea

*These authors contributed equally as co-first authors

^§^These authors contributed equally as co-corresponding authors

**Supplementary figures**

**
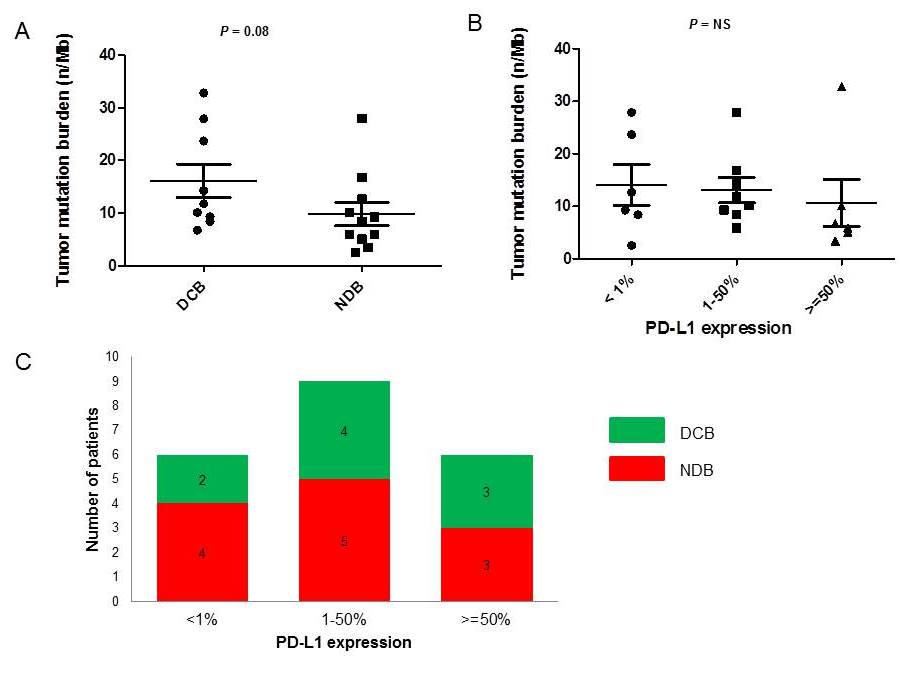
**

**Supplementary figure S1. (A)** Tumor mutation burden (TMB) in patients with durable clinical benefit (DCB) versus non-durable benefit (NDB). Median TMB was 11.76 versus 8.4 mutations per Mb, Mann-Whitney *P*=0.08). **(B)** TMB according to PD-L1 expression. Median TMB was 10.94, 10.93, 6.29/Mb, respectively for the PD-L1 expression of <1%, 1-50%, ≥50%.  **(C)** Distribution of PD-L1 expression (<1%, 1-50%, >=50%) according to clinical outcome. DCB in green and NDB in red.


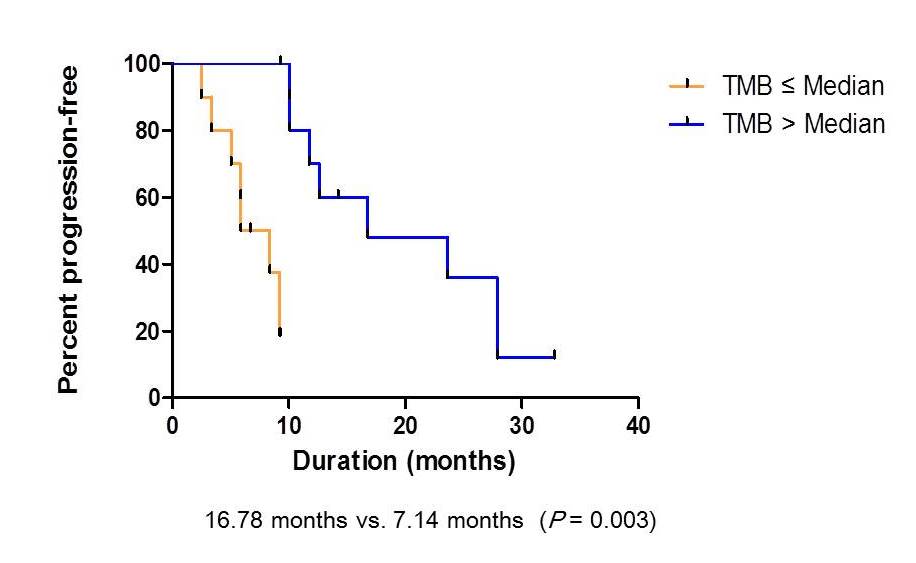


**Supplementary figure S2. Progression-free survival (PFS) in patients according to the median TMB value of 9.27.**Patients with higher TMB showed a prolonged PFS as compared to patients with lower TMB (16.78 months *vs.*7.14 months, *P*=0.003).


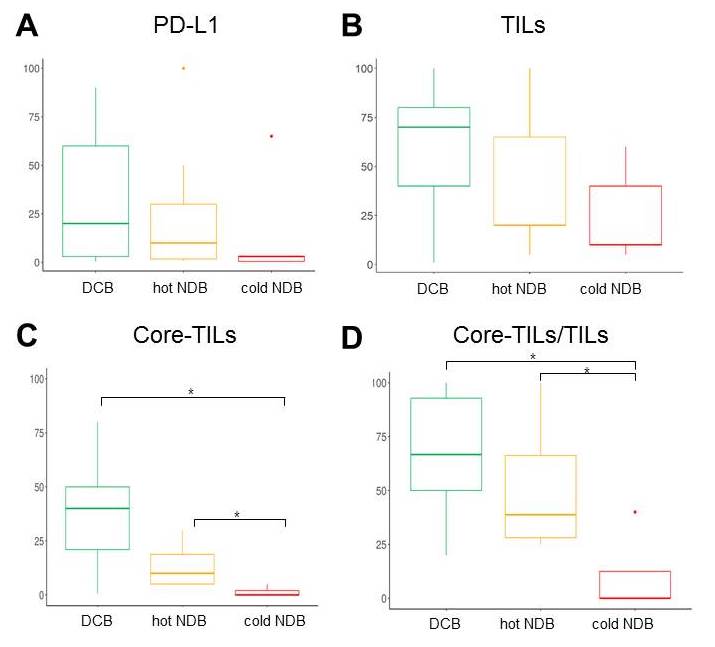


**Supplementary figure S3. PD-L1 expression, tumor-infiltrating lymphocytes (TILs), and core TILs in patients with durable clinical benefit (DCB), non-durable benefit (NDB) with high gene-expression (hot NDB), and NDB with low gene-expression (cold NDB).** **(A)** PD-L1 expression shows no significant difference among three groups, although there is a slight tendency to decrease from DCB to “hot” NDB to “cold” NDB groups. **(B)** There is no significant difference of TIL among three groups. **(C)** For core TIL, DCB group shows significant higher value than two NDB groups (*P*=0.02). There is a significant difference between core TILs of hot and cold NDB groups (*P*=0.04) **(D)** The ratio of core TIL to total TIL was significantly different between the DCB and NDB groups (*P* = 0.03), the “hot” NDB group showed no difference from the DCB group, and the “cold” NDB group showed a lower core TIL ratio than the DCB (*P* = 0.002) and “hot” NDB groups (*P* = 0.03).


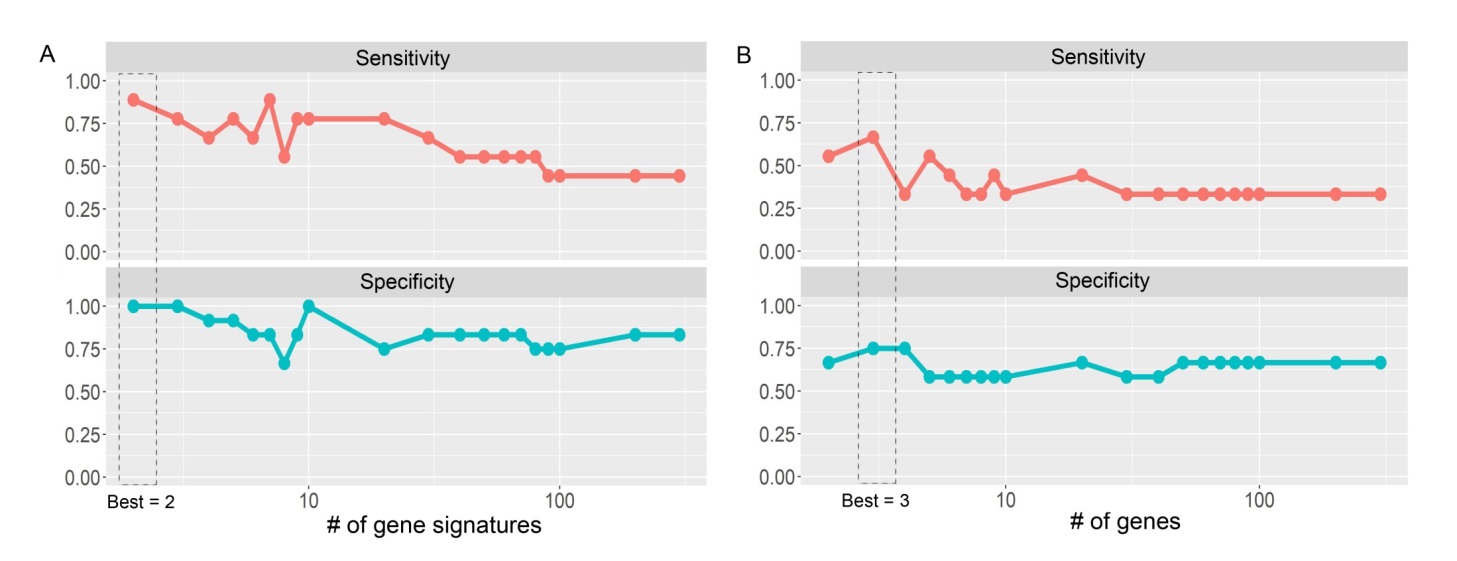


**Supplementary figure S4. The performance of random forest classification models. (A)** Among random forest models of gene signatures, the model of two gene signatures showed the best performance; sensitivity = 0.89 and specificity = 1.0. **(B)** Among random forest models consisted of genes (from two genes to all 393 genes), the model of three genes showed the best performance; sensitivity = 0.67 and specificity = 0.75.


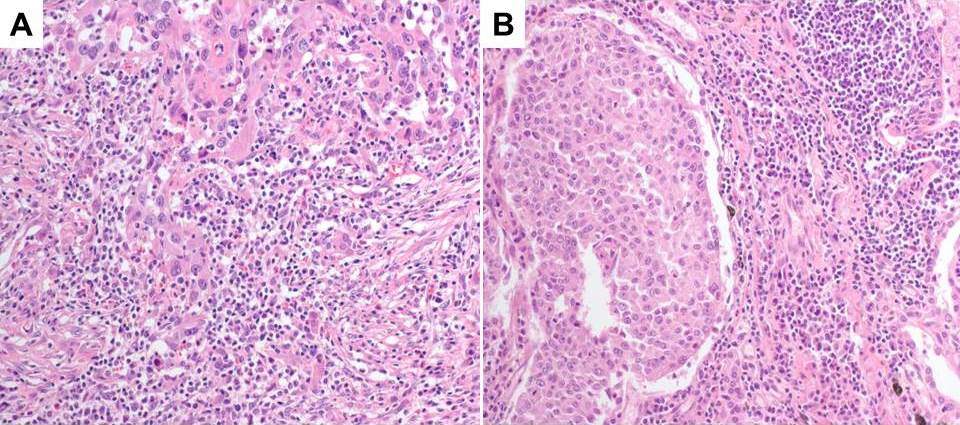


**Supplementary figure S5. A representative picture of tumor immune microenvironment in patients. (A)** Tumor of patient D2 (durable clinical benefit group) shows highly infiltrated lymphocytes within tumor core (Hematoxylin-eosin stain [H-E], x200). **(B)** Tumor of patient N9 (non-durable benefit group with low gene-expression) contains many tumor-infiltrating lymphocytes, but mainly localized along the tumor margin or in fibrotic nests (H-E, x200).

**Supplementary tables**

**Supplementary table S1. The predictability of gene signatures of functional annotation in discriminating between DCB and NDB**

| **ID** | **AUC** | ***t*-test**  **p-value (adjusted)** | **PFS**  **p-value**  **(adjusted)** | **OS**  **p-value**  **(adjusted)** |
| --- | --- | --- | --- | --- |
| **Type II interferon signaling** | 0.77 | 0.0529  (1.0) | 0.917  (1.0) | 0.34  (1.0) |
| **TCR coexpression** | 0.81 | 0.0186  (0.71) | 1.38e-2  (0.57) | 0.76  (1.0) |
| **Neutrophil** | 0.85 | 6.08e-3  (0.24) | 2.26e-2  (0.88) | 0.23  (1.0) |
| **Tumor marker** | 0.85 | 4.83e-3  (0.20) | 0.154  (1.0) | 0.81  (1.0) |
| **Apoptosis** | 0.74 | 0.0279  (1.0) | 0.154  (1.0) | 0.81  (1.0) |

**Supplementary table S2. Description of signatures**

| **Id** | **Description** | **Genes** |
| --- | --- | --- |
| **M1 signature** | Up-regulated genes in classical macrophage 1 compared with alternative macrophage 2 in developmental process (GSE5099, GSE5099 CLASSICAL M1 VS ALTERNATIVE M2 MACROPHAGE UP) | CBLB, CCR7, CD27, CD48, FOXO1, FYB, HLA-B, HLA-G, IFIH1, IKZF4, LAMP3, NFKBIA, SAMHD1 |
| **Peripheral T cell signature** | Down-regulated genes in lymph node T cells compared with fat tissue T cells. (GSE7852, GSE7852 LN VS FAT TCONV UP) | GPR18, HLA-DOA, STAT1 |

**Supplementary table S3. The predictability of selected gene signatures and genes in discriminating DCB and NDB**

| **ID** | **AUC** | ***t*-test**  **p-value** | **edgeR**  **p-value** | **PFS**  **p-value** | **OS**  **p-value** |
| --- | --- | --- | --- | --- | --- |
| **M1 signature** | 1 | 4.95e-4* | 8.54e-11* | 7.84e-5* | 0.348 |
| **Peripheral T cell signature** | 0.944 | 1.08e-4* | 1.45e-10* | 8.29e-3* | 0.695 |
| **BCL2** | 0.87 | 0.122 | 0.558 | 2.68e-3* | 0.408 |
| **TNFRSF9 (CD137)** | 0.93 | 2.78e-3* | 1.23e-3* | 0.0822 | 0.179 |
| **PSMB9** | 0.87 | 6.66e-3* | 0.193 | 4.13e-3* | 0.530 |

*Statistically significant

**Supplementary table S4. The entire list of 395 genes used in the panel**

| Gene | Target | NCBI name | NCBI  ACCESSION | GENE  FUNCTION |
| --- | --- | --- | --- | --- |
| ABCF1 | ABCF1_11741283 | ATP_binding_cassette_subfamily_F_member_1 | NM_001025091 | Housekeeping |
| ADGRE5 | ADGRE5_26552764 | adhesion_G_protein-coupled_receptor_E5 | NM_078481 | Adhesion,migration |
| ADORA2A | ADORA2A_771873 | adenosine_A2a_receptor | NM_000675 | Checkpoint_pathway |
| AIF1 | AIF1_385491 | allograft_inflammatory_factor_1 | NM_001623 | Macrophage |
| AKT1 | AKT1_358467 | AKT_serine_threonine_kinase_1 | NM_001014431 | Tumor_marker |
| ALOX15B | ALOX15B_13391445 | arachidonate_15-lipoxygenase_type_B | NM_001141 | Macrophage |
| ARG1 | ARG1_174278 | arginase_1 | NM_000045 | Myeloid_marker |
| AXL | AXL_24872596 | AXL_receptor_tyrosine_kinase | NM_021913 | Innate_immune_response |
| B3GAT1 | B3GAT1_170280 | beta-13-glucuronyltransferase_1 | NM_018644 | NK_activation |
| BAGE | BAGE_153290 | B_melanoma_antigen | NM_182482 | Tumor_antigen |
| BATF | BATF_237345 | basic_leucine_zipper_ATF-like_transcription_factor | NM_006399 | Helper_T_cells |
| BCL2 | BCL2_10401144 | B-cell_CLL_lymphoma_2 | NM_000633 | Apoptosis |
| BCL2L11 | BCL2L11_620729 | BCL2_like_11 | NM_138621 | Apoptosis |
| BCL6 | BCL6_21502257 | B-cell_CLL_lymphoma_6 | NM_001706 | Type_II_interferon_signaling |
| BRCA1 | BRCA1_42344338 | BRCA1_DNA_repair_associated | NM_007300 | Tumor_marker |
| BRCA2 | BRCA2_98179922 | BRCA2_DNA_repair_associated | NM_000059 | Tumor_marker |
| BST2 | BST2_218322 | bone_marrow_stromal_cell_antigen_2 | NM_004335 | Type_I_interferon_signaling |
| BTLA | BTLA_227334 | B_and_T_lymphocyte_associated | NM_181780 | Checkpoint_pathway |
| BUB1 | BUB1_701803 | BUB1_mitotic_checkpoint_serine_threonine_kinase | NM_004336 | Proliferation |
| C10orf54 | C10orf54_562672 | chromosome_10_open_reading_frame_54 | NM_022153 | Checkpoint_pathway |
| C1QA | C1QA_67171 | complement_component_1_q_subcomponent_A_chain | NM_015991 | Innate_immune_response |
| C1QB | C1QB_111199 | complement_component_1_q_subcomponent_B_chain | NM_000491 | Innate_immune_response |
| CA4 | CA4_420530 | carbonic_anhydrase_4 | NM_000717 | Neutrophil |
| CBLB | CBLB_296407 | Cbl_proto-oncogene_B | NM_170662 | T_cell_receptor_signaling |
| CCL17 | CCL17_288394 | C-C_motif_chemokine_ligand_17 | NM_002987 | Chemokine_signaling |
| CCL18 | CCL18_198296 | C-C_motif_chemokine_ligand_18 | NM_002988 | Lymphocyte_infiltrate |
| CCL2 | CCL2_242346 | C-C_motif_chemokine_ligand_2 | NM_002982 | Lymphocyte_infiltrate |
| CCL20 | CCL20_297403 | C-C_motif_chemokine_ligand_20 | NM_004591 | Chemokine_signaling |
| CCL21 | CCL21_432539 | C-C_motif_chemokine_ligand_21 | NM_002989 | Lymphocyte_infiltrate |
| CCL22 | CCL22_79185 | C-C_motif_chemokine_ligand_22 | NM_002990 | Chemokine_signaling |
| CCL3 | CCL3_238343 | C-C_motif_chemokine_ligand_3 | NM_002983 | Lymphocyte_infiltrate |
| CCL4 | CCL4_121220 | C-C_motif_chemokine_ligand_4 | NM_002984 | Lymphocyte_infiltrate |
| CCL5 | CCL5_110219 | C-C_motif_chemokine_ligand_5 | NM_002985 | Lymphocyte_infiltrate |
| CCNB2 | CCNB2_9861095 | cyclin_B2 | NM_004701 | Proliferation |
| CCR1 | CCR1_54149 | C-C_motif_chemokine_receptor_1 | NM_001295 | Cytokine_signaling |
| CCR2 | CCR2_385483 | C-C_motif_chemokine_receptor_2 | NM_001123396 | Helper_T_cells |
| CCR4 | CCR4_76182 | C-C_motif_chemokine_receptor_4 | NM_005508 | Chemokine_signaling |
| CCR5 | CCR5_85193 | C-C_motif_chemokine_receptor_5_gene_pseudogene | NM_001100168 | Lymphocyte_infiltrate |
| CCR6 | CCR6_271363 | C-C_motif_chemokine_receptor_6 | NM_004367 | Chemokine_signaling |
| CCR7 | CCR7_133237 | C-C_motif_chemokine_receptor_7 | NM_001838 | TCR_coexpression |
| CD14 | CD14_329435 | CD14_molecule | NM_000591 | Dendridic_cell,macrophage |
| CD160 | CD160_194300 | CD160_molecule | NM_007053 | Checkpoint_pathway |
| CD163 | CD163_21422245 | CD163_molecule | NM_004244 | Macrophage |
| CD19 | CD19_12011317 | CD19_molecule | NM_001178098 | B_cell_marker |
| CD1C | CD1C_12531357 | CD1c_molecule | NM_001765 | Antigen_presentation |
| CD1D | CD1D_10661173 | CD1d_molecule | NM_001766 | Antigen_presentation |
| CD2 | CD2_350452 | CD2_molecule | NM_001767 | Lymphocyte_infiltrate |
| CD209 | CD209_9921101 | CD209_molecule | NM_021155 | Dendridic_cell,macrophage |
| CD22 | CD22_10251132 | CD22_molecule | NM_001771 | B_cell_marker |
| CD226 | CD226_9021006 | CD226_molecule | NM_006566 | Adhesion,migration |
| CD244 | CD244_868973 | CD244_molecule | NM_001166663 | Checkpoint_pathway |
| CD247 | CD247_152252 | CD247_molecule | NM_198053 | TCR_coexpression |
| CD27 | CD27_409517 | CD27_molecule | NM_001242 | Drug_target |
| CD274 | CD274_461569 | CD274_molecule | NM_014143 | Checkpoint_pathway |
| CD276 | CD276_12011310 | CD276_molecule | NM_001024736 | Checkpoint_pathway |
| CD28 | CD28_601704 | CD28_molecule | NM_006139 | Checkpoint_pathway |
| CD33 | CD33_387493 | CD33_molecule | NM_001772 | Myeloid_marker |
| CD37 | CD37_496605 | CD37_molecule | NM_001774 | Lymphocyte_infiltrate |
| CD38 | CD38_519628 | CD38_molecule | NM_001775 | Adhesion,migration |
| CD3D | CD3D_400503 | CD3d_molecule | NM_000732 | TCR_coexpression |
| CD3E | CD3E_677780 | CD3e_molecule | NM_000733 | TCR_coexpression |
| CD3G | CD3G_616717 | CD3g_molecule | NM_000073 | TCR_coexpression |
| CD4 | CD4_147255 | CD4_molecule | NM_000616 | Helper_T_cells |
| CD40 | CD40_300407 | CD40_molecule | NM_001250 | Drug_target |
| CD40LG | CD40LG_479579 | CD40_ligand | NM_000074 | T_cell_receptor_signaling |
| CD44 | CD44_10151123 | CD44_molecule_Indian_blood_group | NM_000610 | Adhesion,migration |
| CD47 | CD47_826931 | CD47_molecule | NM_001777 | Adhesion,migration |
| CD48 | CD48_695798 | CD48_molecule | NM_001778 | Checkpoint_pathway |
| CD52 | CD52_70175 | CD52_molecule | NM_001803 | Lymphocyte_infiltrate |
| CD53 | CD53_28133 | CD53_molecule | NM_001040033 | Adhesion,migration |
| CD6 | CD6_17771886 | CD6_molecule | NM_006725 | TCR_coexpression |
| CD63 | CD63_692800 | CD63_molecule | NM_001780 | Lymphocyte_infiltrate |
| CD68 | CD68_172280 | CD68_molecule | NM_001251 | Macrophage |
| CD69 | CD69_195303 | CD69_molecule | NM_001781 | Checkpoint_pathway |
| CD70 | CD70_259363 | CD70_molecule | NM_001252 | Drug_target |
| CD74 | CD74_787896 | CD74_molecule | NM_001025159 | Antigen_processing |
| CD79A | CD79A_184290 | CD79a_molecule | NM_001783 | B_cell_receptor_signaling |
| CD79B | CD79B_484590 | CD79b_molecule | NM_001039933 | B_cell_receptor_signaling |
| CD80 | CD80_457557 | CD80_molecule | NM_005191 | Checkpoint_pathway |
| CD83 | CD83_480580 | CD83_molecule | NM_004233 | Antigen_presentation |
| CD86 | CD86_169267 | CD86_molecule | NM_175862 | Checkpoint_pathway |
| CD8A | CD8A_14271531 | CD8a_molecule | NM_171827 | TCR_coexpression |
| CD8B | CD8B_403510 | CD8b_molecule | NM_172213 | TCR_coexpression |
| CDK1 | CDK1_837939 | cyclin-dependent_kinase_1 | NM_001786 | Proliferation |
| CDKN2A | CDKN2A_696829 | cyclin-dependent_kinase_inhibitor_2A | NM_000077 | Tumor_marker |
| CDKN3 | CDKN3_434534 | cyclin-dependent_kinase_inhibitor_3 | NM_005192 | Proliferation |
| CEACAM1 | CEACAM1_10471155 | carcinoembryonic_antigen_related_cell_adhesion_molecule_1 | NM_001712 | Checkpoint_pathway |
| CEACAM8 | CEACAM8_745847 | carcinoembryonic_antigen_related_cell_adhesion_molecule_8 | NM_001816 | Myeloid_marker |
| CIITA | CIITA_294397 | class_II_major_histocompatibility_complex_transactivator | NM_000246 | Type_II_interferon_signaling |
| CLEC4C | CLEC4C_322429 | C-type_lectin_domain_family_4_member_C | NM_130441 | Dendridic_cell |
| CMKLR1 | CMKLR1_36143 | chemerin_chemokine-like_receptor_1 | NM_001142345 | Dendridic_cell,macrophage |
| CORO1A | CORO1A_457563 | coronin_1A | NM_007074 | Lymphocyte_infiltrate |
| CRTAM | CRTAM_312417 | cytotoxic_and_regulatory_T-cell_molecule | NM_019604 | TCR_coexpression |
| CSF1R | CSF1R_19612067 | colony_stimulating_factor_1_receptor | NM_005211 | Cytokine_signaling |
| CSF2RB | CSF2RB_11971301 | colony_stimulating_factor_2_receptor_beta_common_subunit | NM_000395 | Cytokine_signaling |
| CTAG1B | CTAG1B_276373 | cancer_testis_antigen_1B | NM_001327 | Tumor_antigen |
| CTAG2 | CTAG2_407763 | cancer_testis_antigen_2 | ENST00000369585 | Tumor_antigen |
| CTLA4 | CTLA4_584689 | cytotoxic_T-lymphocyte_associated_protein_4 | NM_005214 | Drug_target |
| CTSS | CTSS_356460 | cathepsin_S | NM_004079 | Lymphocyte_infiltrate |
| CX3CL1 | CX3CL1_190298 | C-X3-C_motif_chemokine_ligand_1 | NM_002996 | Type_II_interferon_signaling |
| CX3CR1 | CX3CR1_35133 | C-X3-C_motif_chemokine_receptor_1 | NM_001337 | Lymphocyte_infiltrate |
| CX3CR1 | CX3CR1_147243 | C-X3-C_motif_chemokine_receptor_1 | NM_001171172 | Lymphocyte_infiltrate |
| CX3CR1 | CX3CR1_207292 | C-X3-C_motif_chemokine_receptor_1 | NM_001171171 | Lymphocyte_infiltrate |
| CX3CR1 | CX3CR1_58187 | C-X3-C_motif_chemokine_receptor_1 | NM_001171174 | Lymphocyte_infiltrate |
| CXCL1 | CXCL1_357459 | C-X-C_motif_chemokine_ligand_1 | NM_001511 | Chemokine_signaling |
| CXCL10 | CXCL10_354459 | C-X-C_motif_chemokine_ligand_10 | NM_001565 | Type_II_interferon_signaling |
| CXCL11 | CXCL11_261361 | C-X-C_motif_chemokine_ligand_11 | NM_005409 | Type_II_interferon_signaling |
| CXCL13 | CXCL13_202307 | C-X-C_motif_chemokine_ligand_13 | NM_006419 | Type_II_interferon_signaling |
| CXCL8 | CXCL8_197302 | C-X-C_motif_chemokine_ligand_8 | NM_000584 | Cytokine_signaling |
| CXCL9 | CXCL9_149250 | C-X-C_motif_chemokine_ligand_9 | NM_002416 | Type_II_interferon_signaling |
| CXCR2 | CXCR2_379486 | C-X-C_motif_chemokine_receptor_2 | NM_001557 | Chemokine_signaling |
| CXCR3 | CXCR3_11138 | C-X-C_motif_chemokine_receptor_3 | NM_001504 | Chemokine_signaling |
| CXCR4 | CXCR4_100208 | C-X-C_motif_chemokine_receptor_4 | NM_003467 | Lymphocyte_infiltrate |
| CXCR5 | CXCR5_153252 | C-X-C_motif_chemokine_receptor_5 | NM_001716 | Type_II_interferon_signaling |
| CXCR6 | CXCR6_48153 | C-X-C_motif_chemokine_receptor_6 | NM_006564 | Lymphocyte_infiltrate |
| CYBB | CYBB_14221529 | cytochrome_b-245_beta_chain | NM_000397 | Type_II_interferon_signaling |
| DDX58 | DDX58_540643 | DEXD_H-box_helicase_58 | NM_014314 | Interferon_signaling |
| DGAT2 | DGAT2_439548 | diacylglycerol_O-acyltransferase_2 | NM_032564 | Neutrophil |
| DMBT1 | DMBT1_64696575 | deleted_in_malignant_brain_tumors_1 | NM_007329 | Innate_immune_response |
| EBI3 | EBI3_527636 | Epstein-Barr_virus_induced_3 | NM_005755 | T_cell_regulation |
| EFNA4 | EFNA4_548649 | ephrin_A4 | NM_005227 | Tumor_marker |
| EGFR | EGFR_31583261 | epidermal_growth_factor_receptor | NM_005228 | Tumor_marker |
| EGR2 | EGR2_454559 | early_growth_response_2 | NM_000399 | T_cell_differentiation |
| EGR3 | EGR3_411516 | early_growth_response_3 | NM_004430 | Tumor_marker |
| EIF2AK2 | EIF2AK2_257357 | eukaryotic_translation_initiation_factor_2_alpha_kinase_2 | NM_001135651 | Type_II_interferon_signaling |
| ENTPD1 | ENTPD1_708815 | ectonucleoside_triphosphate_diphosphohydrolase_1 | NM_001098175 | Checkpoint_pathway |
| EOMES | EOMES_9391044 | eomesodermin | NM_005442 | Checkpoint_pathway |
| FAS | FAS_473575 | Fas_cell_surface_death_receptor | NM_000043 | B_cell_receptor_signaling |
| FASLG | FASLG_417521 | Fas_ligand | NM_000639 | Type_II_interferon_signaling |
| FCER1G | FCER1G_31138 | Fc_fragment_of_IgE_receptor_Ig | NM_004106 | Lymphocyte_infiltrate |
| FCGR1A | FCGR1A_547652 | Fc_fragment_of_IgG_receptor_Ia | NM_000566 | B_cell_marker |
| FCGR2B | FCGR2B_792903 | Fc_fragment_of_IgG_receptor_IIb | NM_004001 | B_cell_marker |
| FCGR3A | FCGR3A_575682 | Fc_fragment_of_IgG_receptor_IIIa | NM_000569 | Macrophage |
| FCGR3B | FCGR3B_620722 | Fc_fragment_of_IgG_receptor_IIIb | NM_000570 | NK_activation |
| FCRLA | FCRLA_710815 | Fc_receptor_like_A | NM_001184866 | B_cell_marker |
| FOXM1 | FOXM1_10551166 | forkhead_box_M1 | NM_021953 | Proliferation |
| FOXO1 | FOXO1_9761081 | forkhead_box_O1 | NM_002015 | PD-1_signaling,tumor_marker |
| FOXP3 | FOXP3_865975 | forkhead_box_P3 | NM_014009 | T_cell_regulation |
| FUT4 | FUT4_31443252 | fucosyltransferase_4 | NM_002033 | Myeloid_marker,stem_cell |
| FYB | FYB_12891393 | FYN_binding_protein | NM_001465 | Lymphocyte_infiltrate |
| G6PD | G6PD_10841194 | glucose-6-phosphate_dehydrogenase | NM_000402 | Housekeeping |
| GADD45GIP1 | GADD45GIP1_329429 | GADD45G_interacting_protein_1 | NM_052850 | Apoptosis |
| GAGE1,GAGE12I,GAGE12F | GAGE1_144260 | G_antigen_1 | NM_001468,NM_001477,NM_001098405 | Tumor_antigen |
| GAGE10 | GAGE10_137250 | G_antigen_10 | NM_001098413 | Tumor_antigen |
| GAGE12J | GAGE12J_144260 | G_antigen_12J | NM_001098406 | Tumor_antigen |
| GAGE13 | GAGE13_116232 | G_antigen_13 | NM_001098412 | Tumor_antigen |
| GAGE2C,GAGE2A,GAGE2E | GAGE2C_104217 | G_antigen_2C | NM_001472,NM_001127212,NM_001127200 | Tumor_antigen |
| GATA3 | GATA3_14061514 | GATA_binding_protein_3 | NM_001002295 | Helper_T_cells |
| GBP1 | GBP1_771872 | guanylate_binding_protein_1 | NM_002053 | Type_II_interferon_signaling |
| GNLY | GNLY_251357 | granulysin | NM_006433 | NK_activation |
| GPR18 | GPR18_238340 | G_protein-coupled_receptor_18 | NM_001098200 | TCR_coexpression |
| GRAP2 | GRAP2_283387 | GRB2-related_adaptor_protein_2 | NM_004810 | TCR_coexpression |
| GUSB | GUSB_17671872 | glucuronidase_beta | NM_000181 | Housekeeping |
| GZMA | GZMA_165265 | granzyme_A | NM_006144 | Lymphocyte_infiltrate |
| GZMB | GZMB_581688 | granzyme_B | NM_004131 | Lymphocyte_infiltrate |
| GZMH | GZMH_233339 | granzyme_H | NM_033423 | Lymphocyte_infiltrate |
| GZMK | GZMK_178286 | granzyme_K | NM_002104 | Lymphocyte_infiltrate |
| HAVCR2 | HAVCR2_594705 | hepatitis_A_virus_cellular_receptor_2 | NM_032782 | Checkpoint_pathway |
| HERC6 | HERC6_17021806 | HECT_and_RLD_domain_containing_E3_ubiquitin_protein_ligase_family_member_6 | NM_017912 | Dendridic_cell |
| HGF | HGF_14151518 | hepatocyte_growth_factor | NM_000601 | Cytokine_signaling |
| HIF1A | HIF1A_13371439 | hypoxia_inducible_factor_1_alpha_subunit | NM_001530 | PD-1_signaling,tumor_marker |
| HLA-A | HLA-A_345445 | major_histocompatibility_complex_class_I_A | NM_002116 | Antigen_processing |
| HLA-B | HLA-B_639746 | major_histocompatibility_complex_class_I_B | NM_005514 | Antigen_processing |
| HLA-C | HLA-C_9191023 | major_histocompatibility_complex_class_I_C | NM_002117 | Antigen_processing |
| HLA-DMA | HLA-DMA_764869 | major_histocompatibility_complex_class_II_DM_alpha | NM_006120 | Antigen_processing |
| HLA-DMB | HLA-DMB_854962 | major_histocompatibility_complex_class_II_DM_beta | NM_002118 | Antigen_processing |
| HLA-DOA | HLA-DOA_55163 | major_histocompatibility_complex_class_II_DO_alpha | NM_002119 | Antigen_processing |
| HLA-DOB | HLA-DOB_182290 | major_histocompatibility_complex_class_II_DO_beta | NM_002120 | Antigen_processing |
| HLA-DPA1 | HLA-DPA1_129237 | major_histocompatibility_complex_class_II_DP_alpha_1 | NM_033554 | Antigen_processing |
| HLA-DPB1 | HLA-DPB1_175280 | major_histocompatibility_complex_class_II_DP_beta_1 | NM_002121 | Antigen_processing |
| HLA-DQA1 | HLA-DQA1_587686 | major_histocompatibility_complex_class_II_DQ_alpha_1 | NM_002122 | Antigen_processing |
| HLA-DQA2 | HLA-DQA2_840937 | major_histocompatibility_complex_class_II_DQ_alpha_2 | NM_020056 | Antigen_processing |
| HLA-DQB2 | HLA-DQB2_142243 | major_histocompatibility_complex_class_II_DQ_beta_2 | NM_001198858 | Antigen_processing |
| HLA-DRA | HLA-DRA_186294 | major_histocompatibility_complex_class_II_DR_alpha | NM_019111 | Antigen_processing |
| HLA-DRB1 | HLA-DRB1_877982 | major_histocompatibility_complex_class_II_DR_beta_1 | NM_002124 | Antigen_processing |
| HLA-E | HLA-E_639747 | major_histocompatibility_complex_class_I_E | NM_005516 | Antigen_processing |
| HLA-F | HLA-F_101204 | major_histocompatibility_complex_class_I_F | NM_001098479 | Antigen_processing |
| HLA-F-AS1 | HLA-F-AS1_726834 | HLA-F_antisense_RNA_1 | NR_026972 | Antigen_processing |
| HLA-G | HLA-G_483585 | major_histocompatibility_complex_class_I_G | NM_002127 | Antigen_processing |
| HMBS | HMBS_796904 | hydroxymethylbilane_synthase | NM_000190 | Housekeeping |
| ICAM1 | ICAM1_605711 | intercellular_adhesion_molecule_1 | NM_000201 | Type_II_interferon_signaling |
| ICOS | ICOS_382487 | inducible_T-cell_costimulator | NM_012092 | Checkpoint_pathway |
| ICOSLG | ICOSLG_483588 | inducible_T-cell_costimulator_ligand | NM_015259 | Checkpoint_pathway |
| ID2 | ID2_579679 | inhibitor_of_DNA_binding_2_HLH_protein | NM_002166 | T_cell_regulation |
| ID3 | ID3_572678 | inhibitor_of_DNA_binding_3_HLH_protein | NM_002167 | T_cell_regulation |
| IDO1 | IDO1_268369 | indoleamine_23-dioxygenase_1 | NM_002164 | Drug_target |
| IDO2 | IDO2_458562 | indoleamine_23-dioxygenase_2 | NM_194294 | Checkpoint_pathway |
| IFI27 | IFI27_37143 | interferon_alpha_inducible_protein_27 | NM_005532 | Type_I_interferon_signaling |
| IFI35 | IFI35_419526 | interferon_induced_protein_35 | NM_005533 | Interferon_signaling |
| IFI44L | IFI44L_12771376 | interferon_induced_protein_44_like | NM_006820 | Interferon_signaling |
| IFI6 | IFI6_47156 | interferon_alpha_inducible_protein_6 | NM_022873 | Interferon_signaling |
| IFIH1 | IFIH1_20172123 | interferon_induced_with_helicase_C_domain_1 | NM_022168 | Innate_immune_response |
| IFIT1 | IFIT1_158259 | interferon_induced_protein_with_tetratricopeptide_repeats_1 | NM_001548 | Type_I_interferon_signaling |
| IFIT2 | IFIT2_124224 | interferon_induced_protein_with_tetratricopeptide_repeats_2 | NM_001547 | Cytokine_signaling |
| IFIT3 | IFIT3_72174 | interferon_induced_protein_with_tetratricopeptide_repeats_3 | NM_001031683 | Type_I_interferon_signaling |
| IFITM1 | IFITM1_359459 | interferon_induced_transmembrane_protein_1 | NM_003641 | Type_I_interferon_signaling |
| IFITM2 | IFITM2_316422 | interferon_induced_transmembrane_protein_2 | NM_006435 | Type_I_interferon_signaling |
| IFNA17 | IFNA17_691788 | interferon_alpha_17 | NM_021268 | T_cell_receptor_signaling |
| IFNB1 | IFNB1_579686 | interferon_beta_1 | NM_002176 | Type_II_interferon_signaling |
| IFNG | IFNG_400500 | interferon_gamma | NM_000619 | Type_II_interferon_signaling |
| IGF1R | IGF1R_12291338 | insulin_like_growth_factor_1_receptor | NM_000875 | Adhesion,migration |
| IGSF6 | IGSF6_411515 | immunoglobulin_superfamily_member_6 | NM_005849 | Lymphocyte_infiltrate |
| IKZF1 | IKZF1_189301 | IKAROS_family_zinc_finger_1 | NM_006060 | Lymphocyte_development |
| IKZF2 | IKZF2_537639 | IKAROS_family_zinc_finger_2 | NM_016260 | Lymphocyte_development |
| IKZF3 | IKZF3_427534 | IKAROS_family_zinc_finger_3 | NM_012481 | TCR_coexpression |
| IKZF4 | IKZF4_746852 | IKAROS_family_zinc_finger_4 | NM_022465 | Lymphocyte_development |
| IL10 | IL10_491598 | interleukin_10 | NM_000572 | Drug_target |
| IL10RA | IL10RA_192296 | interleukin_10_receptor_subunit_alpha | NM_001558 | Lymphocyte_infiltrate |
| IL12A | IL12A_739842 | interleukin_12A | NM_000882 | Drug_target |
| IL12B | IL12B_314422 | interleukin_12B | NM_002187 | Drug_target |
| IL13 | IL13_268378 | interleukin_13 | NM_002188 | Cytokine_signaling |
| IL15 | IL15_49155 | interleukin_15 | NM_000585 | T_cell_regulation |
| IL17A | IL17A_233320 | interleukin_17A | NM_002190 | Helper_T_cells |
| IL17F | IL17F_35139 | interleukin_17F | NM_052872 | Dendridic_cell,macrophage |
| IL18 | IL18_140246 | interleukin_18 | NM_001562 | T_cell_regulation |
| IL1A | IL1A_14431546 | interleukin_1_alpha | NM_000575 | Cytokine_signaling |
| IL1B | IL1B_659766 | interleukin_1_beta | NM_000576 | Type_II_interferon_signaling |
| IL2 | IL2_366451 | interleukin_2 | NM_000586 | Drug_target |
| IL21 | IL21_368450 | interleukin_21 | NM_021803 | Cytokine_signaling |
| IL22 | IL22_263349 | interleukin_22 | NM_020525 | T_cell_regulation |
| IL23A | IL23A_386491 | interleukin_23_subunit_alpha | NM_016584 | Dendridic_cell,macrophage |
| IL2RA | IL2RA_240338 | interleukin_2_receptor_subunit_alpha | NM_000417 | Cytokine_signaling |
| IL2RB | IL2RB_9991107 | interleukin_2_receptor_subunit_beta | NM_000878 | TCR_coexpression |
| IL2RG | IL2RG_884988 | interleukin_2_receptor_subunit_gamma | NM_000206 | Lymphocyte_infiltrate |
| IL3RA | IL3RA_465565 | interleukin_3_receptor_subunit_alpha | NM_002183 | Dendridic_cell |
| IL4 | IL4_380477 | interleukin_4 | NM_000589 | Cytokine_signaling |
| IL6 | IL6_521626 | interleukin_6 | NM_000600 | Cytokine_signaling |
| IL7 | IL7_545652 | interleukin_7 | NM_000880 | Cytokine_signaling |
| IL7R | IL7R_551658 | interleukin_7_receptor | NM_002185 | TCR_coexpression |
| IRF1 | IRF1_752861 | interferon_regulatory_factor_1 | NM_002198 | Type_II_interferon_signaling |
| IRF4 | IRF4_786895 | interferon_regulatory_factor_4 | NM_002460 | Interferon_signaling |
| IRF9 | IRF9_12191322 | interferon_regulatory_factor_9 | NM_006084 | Type_II_interferon_signaling |
| IRS1 | IRS1_37283828 | insulin_receptor_substrate_1 | NM_005544 | Tumor_marker |
| ISG15 | ISG15_66173 | ISG15_ubiquitin-like_modifier | NM_005101 | Type_I_interferon_signaling |
| ISG20 | ISG20_642750 | interferon_stimulated_exonuclease_gene_20 | NM_002201 | Type_I_interferon_signaling |
| ITGA1 | ITGA1_814914 | integrin_subunit_alpha_1 | NM_181501 | Adhesion,migration |
| ITGAE | ITGAE_33753481 | integrin_subunit_alpha_E | NM_002208 | Adhesion,migration |
| ITGAL | ITGAL_535638 | integrin_subunit_alpha_L | NM_002209 | Leukocyte_migration |
| ITGAM | ITGAM_31033209 | integrin_subunit_alpha_M | NM_001145808 | Leukocyte_migration |
| ITGAX | ITGAX_20482151 | integrin_subunit_alpha_X | NM_000887 | Dendridic_cell |
| ITGB1 | ITGB1_679777 | integrin_subunit_beta_1 | NM_002211 | Adhesion,migration |
| ITGB2 | ITGB2_15421648 | integrin_subunit_beta_2 | NM_000211 | Lymphocyte_infiltrate |
| ITGB7 | ITGB7_24342544 | integrin_subunit_beta_7 | NM_000889 | Leukocyte_migration |
| ITK | ITK_563669 | IL2_inducible_T-cell_kinase | NM_005546 | TCR_coexpression |
| JAML | JAML_78188 | junction_adhesion_molecule_like | NM_001098526 | Lymphocyte_infiltrate |
| JCHAIN | JCHAIN_134238 | joining_chain_of_multimeric_IgA_and_IgM | NM_144646 | B_cell_marker |
| KIAA0101 | KIAA0101_319426 | KIAA0101 | NM_014736 | Proliferation |
| KIR2DL1 | KIR2DL1_9142 | killer_cell_immunoglobulin_like_receptor_two_Ig_domains_and_long_cytoplasmic_tail_1 | NM_014218 | Drug_target |
| KIR2DL2 | KIR2DL2_27126 | killer_cell_immunoglobulin_like_receptor_two_Ig_domains_and_long_cytoplasmic_tail_2 | NM_014218 | NK_cell_marker |
| KIR2DL3 | KIR2DL3_654757 | killer_cell_immunoglobulin_like_receptor_two_Ig_domains_and_long_cytoplasmic_tail_3 | NM_015868 | NK_cell_marker |
| KLF2 | KLF2_9111017 | Kruppel_like_factor_2 | NM_016270 | T_cell_regulation,trafficking |
| KLRB1 | KLRB1_177284 | killer_cell_lectin_like_receptor_B1 | NM_002258 | NK_activation |
| KLRD1 | KLRD1_515619 | killer_cell_lectin_like_receptor_D1 | NM_002262 | Drug_target |
| KLRF1 | KLRF1_188285 | killer_cell_lectin_like_receptor_F1 | NM_016523 | NK_activation |
| KLRG1 | KLRG1_410518 | killer_cell_lectin_like_receptor_G1 | NM_005810 | NK_activation |
| KLRK1 | KLRK1_698804 | killer_cell_lectin_like_receptor_K1 | NM_007360 | NK_activation |
| KREMEN1 | KREMEN1_12861389 | kringle_containing_transmembrane_protein_1 | NM_032045 | Neutrophil |
| KRT5 | KRT5_10631165 | keratin_5 | NM_000424 | Tumor_marker |
| KRT7 | KRT7_440543 | keratin_7 | NM_005556 | Tumor_marker |
| LAG3 | LAG3_13111419 | lymphocyte_activating_3 | NM_002286 | Drug_target |
| LAMP1 | LAMP1_374485 | lysosomal_associated_membrane_protein_1 | NM_005561 | Lymphocyte_infiltrate |
| LAMP3 | LAMP3_12361344 | lysosomal_associated_membrane_protein_3 | NM_014398 | TCR_coexpression |
| LAPTM5 | LAPTM5_555664 | lysosomal_protein_transmembrane_5 | NM_006762 | Lymphocyte_infiltrate |
| LCK | LCK_89192 | LCK_proto-oncogene_Src_family_tyrosine_kinase | NM_001042771 | TCR_coexpression |
| LCN2 | LCN2_153259 | lipocalin_2 | NM_005564 | Innate_immune_response |
| LEXM | LEXM_10551156 | lymphocyte_expansion_molecule | NM_001110533 | T_cell_differentiation |
| LILRB1 | LILRB1_15681666 | leukocyte_immunoglobulin_like_receptor_B1 | NM_001081637 | Leukocyte_inhibition |
| LILRB2 | LILRB2_891994 | leukocyte_immunoglobulin_like_receptor_B2 | NM_001080978 | Lymphocyte_infiltrate |
| LMNA | LMNA_588696 | lamin_A_C | NM_170707 | Housekeeping |
| LRG1 | LRG1_24131 | leucine_rich_alpha-2-glycoprotein_1 | NM_052972 | Neutrophil |
| LRP1 | LRP1_25642670 | LDL_receptor_related_protein_1 | NM_002332 | Housekeeping |
| LST1 | LST1_22120 | leukocyte_specific_transcript_1 | NM_007161 | Leukocyte_inhibition |
| LY9 | LY9_13751476 | lymphocyte_antigen_9 | NM_002348 | Lymphocyte_infiltrate |
| LYZ | LYZ_120222 | lysozyme | NM_000239 | Innate_immune_response |
| M6PR | M6PR_229336 | mannose-6-phosphate_receptor_cation_dependent | NM_002355 | T_cell_regulation |
| MAD2L1 | MAD2L1_115221 | MAD2_mitotic_arrest_deficient-like_1_yeast | NM_002358 | Proliferation |
| MADCAM1 | MADCAM1_9291024 | mucosal_vascular_addressin_cell_adhesion_molecule_1 | NM_130760 | Adhesion,migration |
| MAGEA1 | MAGEA1_22129 | MAGE_family_member_A1 | NM_004988 | Tumor_antigen |
| MAGEA10 | MAGEA10_241320 | MAGE_family_member_A10 | NM_021048 | Tumor_antigen |
| MAGEA12 | MAGEA12_30130 | MAGE_family_member_A12 | NM_005367 | Tumor_antigen |
| MAGEA3 | MAGEA3_20121 | MAGE_family_member_A3 | NM_005362,NM_005363 | Tumor_antigen |
| MAGEA4 | MAGEA4_28131 | MAGE_family_member_A4 | NM_001011548 | Tumor_antigen |
| MAGEC2 | MAGEC2_249358 | MAGE_family_member_C2 | NM_016249 | Tumor_antigen |
| MAPK1 | MAPK1_13241425 | mitogen-activated_protein_kinase_1 | NM_002745 | Tumor_marker |
| MAPK14 | MAPK14_14031511 | mitogen-activated_protein_kinase_14 | NM_139012 | Innate_immune_response |
| MELK | MELK_300401 | maternal_embryonic_leucine_zipper_kinase | NM_014791 | Proliferation |
| MIF | MIF_157270 | macrophage_migration_inhibitory_factor_glycosylation-inhibiting_factor | NM_002415 | Innate_immune_response |
| MKI67 | MKI67_581686 | marker_of_proliferation_Ki-67 | NM_002417 | Proliferation |
| MLANA | MLANA_159265 | melan-A | NM_005511 | Tumor_antigen |
| MMP2 | MMP2_11051213 | matrix_metallopeptidase_2 | NM_004530 | Tumor_marker |
| MMP9 | MMP9_11741283 | matrix_metallopeptidase_9 | NM_004994 | Tumor_marker |
| MPO | MPO_15121620 | myeloperoxidase | NM_000250 | Myeloid_marker |
| MRC1 | MRC1_529634 | mannose_receptor_C_type_1 | NM_002438 | Dendridic_cell,macrophage |
| MS4A1 | MS4A1_127232 | membrane_spanning_4-domains_A1 | NM_021950 | Drug_target |
| MTOR | MTOR_62366338 | mechanistic_target_of_rapamycin | NM_004958 | PD-1_signaling,tumor_marker |
| MX1 | MX1_232336 | MX_dynamin_like_GTPase_1 | NM_001178046 | Interferon_signaling |
| MYC | MYC_510619 | v-myc_avian_myelocytomatosis_viral_oncogene_homolog | NM_002467 | Tumor_marker |
| NCAM1 | NCAM1_26012709 | neural_cell_adhesion_molecule_1 | NM_181351 | Adhesion,migration |
| NCF1 | NCF1_509617 | neutrophil_cytosolic_factor_1 | NM_000265 | Chemokine_signaling |
| NCR1 | NCR1_754861 | natural_cytotoxicity_triggering_receptor_1 | NM_004829 | NK_cell_marker |
| NCR3 | NCR3_234342 | natural_cytotoxicity_triggering_receptor_3 | NM_147130 | NK_cell_marker |
| NECTIN2 | NECTIN2_13881496 | nectin_cell_adhesion_molecule_2 | NM_001042724 | Adhesion,migration |
| NFATC1 | NFATC1_27682875 | nuclear_factor_of_activated_T-cells_1 | NM_172387 | PD-1_signaling |
| NFKBIA | NFKBIA_734840 | NFKB_inhibitor_alpha | NM_020529 | T_cell_receptor_signaling |
| NKG7 | NKG7_557664 | natural_killer_cell_granule_protein_7 | NM_005601 | Lymphocyte_infiltrate |
| NOS2 | NOS2_91195 | nitric_oxide_synthase_2 | NM_000625 | Innate_immune_response |
| NOTCH3 | NOTCH3_738847 | notch_3 | NM_000435 | Tumor_marker |
| NRP1 | NRP1_14831588 | neuropilin_1 | NM_003873 | Dendridic_cell |
| NT5E | NT5E_12531355 | 5-nucleotidase_ecto | NM_002526 | Checkpoint_pathway |
| NTN3 | NTN3_12821377 | netrin_3 | NM_006181 | B_cell_marker |
| OAS1 | OAS1_757865 | 2-5-oligoadenylate_synthetase_1 | NM_016816 | Type_II_interferon_signaling |
| OAS2 | OAS2_15981707 | 2-5-oligoadenylate_synthetase_2 | NM_016817 | Interferon_signaling |
| OAS3 | OAS3_667776 | 2-5-oligoadenylate_synthetase_3 | NM_006187 | Interferon_signaling |
| PDCD1 | PDCD1_143244 | programmed_cell_death_1 | NM_005018 | Drug_target |
| PDCD1LG2 | PDCD1LG2_315423 | programmed_cell_death_1_ligand_2 | NM_025239 | Checkpoint_pathway |
| PECAM1 | PECAM1_22132 | platelet_and_endothelial_cell_adhesion_molecule_1 | NM_000442 | Adhesion,migration |
| PGF | PGF_742842 | placental_growth_factor | NM_002632 | Tumor_marker |
| PIK3CA | PIK3CA_12781384 | phosphatidylinositol-45-bisphosphate_3-kinase_catalytic_subunit_alpha | NM_006218 | PD-1_signaling,tumor_marker |
| PIK3CD | PIK3CD_19842095 | phosphatidylinositol-45-bisphosphate_3-kinase_catalytic_subunit_delta | NM_005026 | PD-1_signaling,tumor_marker |
| PMEL | PMEL_233339 | premelanosome_protein | NM_006928 | Drug_target |
| POLR2A | POLR2A_42334334 | polymerase_RNA_II_subunit_A | NM_000937 | Housekeeping |
| POU2AF1 | POU2AF1_230337 | POU_class_2_associating_factor_1 | NM_006235 | B_cell_marker |
| PRDM1 | PRDM1_484588 | PR_domain_1 | NM_001198 | PD-1_signaling |
| PRF1 | PRF1_672778 | perforin_1 | NM_005041 | NK_activation |
| PSMB9 | PSMB9_384491 | proteasome_subunit_beta_9 | NM_002800 | Type_II_interferon_signaling |
| PTEN | PTEN_19732078 | phosphatase_and_tensin_homolog | NM_000314 | PD-1_signaling,tumor_marker |
| PTGS2 | PTGS2_14761583 | prostaglandin-endoperoxide_synthase_2 | NM_000963 | Tumor_marker |
| PTK7 | PTK7_15161625 | protein_tyrosine_kinase_7_inactive | NM_002821 | Tumor_marker |
| PTPN11 | PTPN11_14261533 | protein_tyrosine_phosphatase_non-receptor_type_11 | NM_002834 | PD-1_signaling,tumor_marker |
| PTPN6 | PTPN6_17741884 | protein_tyrosine_phosphatase_non-receptor_type_6 | NM_080548 | T_cell_receptor_signaling |
| PTPN7 | PTPN7_471581 | protein_tyrosine_phosphatase_non-receptor_type_7 | NM_001199797 | Lymphocyte_infiltrate |
| PTPRC | PTPRC_710817 | protein_tyrosine_phosphatase_receptor_type_C | NM_002838 | Lymphocyte_infiltrate |
| PTPRCAP | PTPRCAP_383516 | protein_tyrosine_phosphatase_receptor_type_C_associated_protein | NM_005608 | TCR_coexpression |
| PVR | PVR_9571064 | poliovirus_receptor | NM_006505 | Checkpoint_pathway |
| PYGL | PYGL_586697 | phosphorylase_glycogen_liver | NM_002863 | Neutrophil |
| RB1 | RB1_24592560 | RB_transcriptional_corepressor_1 | NM_000321 | Tumor_marker |
| RORC | RORC_12011307 | RAR_related_orphan_receptor_C | NM_005060 | Helper_T_cells |
| RPS6 | RPS6_124229 | ribosomal_protein_S6 | NM_001010 | Tumor_marker |
| S100A8 | S100A8_239334 | S100_calcium_binding_protein_A8 | NM_002964 | Myeloid_marker,MDSC |
| S100A9 | S100A9_174280 | S100_calcium_binding_protein_A9 | NM_002965 | Myeloid_marker,MDSC |
| SAMHD1 | SAMHD1_19002006 | SAM_and_HD_domain_containing_deoxynucleoside_triphosphate_triphosphohydrolase_1 | NM_015474 | Lymphocyte_infiltrate |
| SDHA | SDHA_16361735 | succinate_dehydrogenase_complex_flavoprotein_subunit_A | NM_004168 | Housekeeping |
| SELL | SELL_139245 | selectin_L | NM_000655 | Leukocyte_migration |
| SH2D1A | SH2D1A_540642 | SH2_domain_containing_1A | NM_002351 | Lymphocyte_activation |
| SH2D1B | SH2D1B_173275 | SH2_domain_containing_1B | NM_053282 | Lymphocyte_activation |
| SIT1 | SIT1_148254 | signaling_threshold_regulating_transmembrane_adaptor_1 | NM_014450 | Lymphocyte_infiltrate |
| SKAP2 | SKAP2_809912 | src_kinase_associated_phosphoprotein_2 | NM_003930 | B_cell_marker |
| SLAMF7 | SLAMF7_9161020 | SLAM_family_member_7 | NM_021181 | Drug_target |
| SLAMF8 | SLAMF8_9071016 | SLAM_family_member_8 | NM_020125 | Lymphocyte_infiltrate |
| SNAI1 | SNAI1_138246 | snail_family_transcriptional_repressor_1 | NM_005985 | Tumor_marker,stemness |
| SNAI2 | SNAI2_722828 | snail_family_transcriptional_repressor_2 | NM_003068 | Tumor_marker,stemness |
| SRGN | SRGN_230328 | serglycin | NM_002727 | Lymphocyte_infiltrate |
| SSX2 | SSX2_233349 | SSX_family_member_2 | NM_003147 | Tumor_antigen |
| STAT1 | STAT1_18871996 | signal_transducer_and_activator_of_transcription_1 | NM_007315 | Type_II_interferon_signaling |
| STAT3 | STAT3_17511856 | signal_transducer_and_activator_of_transcription_3 | NM_139276 | Drug_target |
| STAT4 | STAT4_245352 | signal_transducer_and_activator_of_transcription_4 | NM_003151 | Helper_T_cells |
| STAT5A | STAT5A_18501954 | signal_transducer_and_activator_of_transcription_5A | NM_003152 | Cytokine_signaling |
| STAT6 | STAT6_231339 | signal_transducer_and_activator_of_transcription_6 | NM_003153 | Helper_T_cells |
| TAGAP | TAGAP_10531160 | T-cell_activation_RhoGTPase_activating_protein | NM_054114 | Lymphocyte_infiltrate |
| TAP1 | TAP1_15701675 | transporter_1_ATP-binding_cassette_sub-family_B_MDR_TAP | NM_000593 | Type_II_interferon_signaling |
| TARP | TARP_416517 | TCR_gamma_alternate_reading_frame_protein | NM_001003806 | Lymphocyte_infiltrate |
| TBP | TBP_259367 | TATA-box_binding_protein | NM_003194 | Housekeeping |
| TBX21 | TBX21_9251036 | T-box_21 | NM_013351 | Type_II_interferon_signaling |
| TCF7 | TCF7_677799 | transcription_factor_7_T-cell_specific_HMG-box | NM_003202 | Tumor_marker |
| TDO2 | TDO2_55162 | tryptophan_23-dioxygenase | NM_005651 | Checkpoint_pathway |
| TFRC | TFRC_10891196 | transferrin_receptor | NM_001128148 | Housekeeping |
| TGFB1 | TGFB1_13681474 | transforming_growth_factor_beta_1 | NM_000660 | Checkpoint_pathway |
| TIGIT | TIGIT_383491 | T-cell_immunoreceptor_with_Ig_and_ITIM_domains | NM_173799 | TCR_coexpression |
| TLR3 | TLR3_24932595 | toll_like_receptor_3 | NM_003265 | Dendridic_cell |
| TLR7 | TLR7_99206 | toll_like_receptor_7 | NM_016562 | Innate_immune_response |
| TLR8 | TLR8_36137 | toll_like_receptor_8 | NM_138636 | Lymphocyte_infiltrate |
| TLR9 | TLR9_619727 | toll_like_receptor_9 | NM_017442 | Drug_target |
| TNF | TNF_450555 | tumor_necrosis_factor | NM_000594 | Checkpoint_pathway |
| TNFAIP8 | TNFAIP8_188295 | TNF_alpha_induced_protein_8 | NM_014350 | Lymphocyte_infiltrate |
| TNFRSF14 | TNFRSF14_846950 | tumor_necrosis_factor_receptor_superfamily_member_14 | NM_003820 | Checkpoint_pathway |
| TNFRSF17 | TNFRSF17_254359 | tumor_necrosis_factor_receptor_superfamily_member_17 | NM_001192 | B_cell_marker |
| TNFRSF18 | TNFRSF18_348456 | tumor_necrosis_factor_receptor_superfamily_member_18 | NM_004195 | Drug_target |
| TNFRSF4 | TNFRSF4_304389 | tumor_necrosis_factor_receptor_superfamily_member_4 | NM_003327 | Drug_target |
| TNFRSF9 | TNFRSF9_894998 | tumor_necrosis_factor_receptor_superfamily_member_9 | NM_001561 | Drug_target |
| TNFSF10 | TNFSF10_251357 | tumor_necrosis_factor_superfamily_member_10 | NM_003810 | Apoptosis |
| TNFSF13B | TNFSF13B_774879 | tumor_necrosis_factor_superfamily_member_13b | NM_006573 | B_cell_marker |
| TNFSF14 | TNFSF14_224334 | tumor_necrosis_factor_superfamily_member_14 | NM_003807 | Checkpoint_pathway |
| TNFSF18 | TNFSF18_128228 | tumor_necrosis_factor_superfamily_member_18 | NM_005092 | Checkpoint_pathway |
| TNFSF4 | TNFSF4_347439 | tumor_necrosis_factor_superfamily_member_4 | NM_003326 | Checkpoint_pathway |
| TNFSF9 | TNFSF9_325412 | tumor_necrosis_factor_superfamily_member_9 | NM_003811 | Cytokine_signaling |
| TOP2A | TOP2A_27522855 | topoisomerase_DNA_II_alpha | NM_001067 | Proliferation |
| TP63 | TP63_143246 | tumor_protein_p63 | NM_003722 | Tumor_marker |
| TRIM29 | TRIM29_882990 | tripartite_motif_containing_29 | NM_012101 | Tumor_marker |
| TUBB | TUBB_369479 | tubulin_beta_class_I | NM_178014 | Housekeeping |
| TWIST1 | TWIST1_9291033 | twist_family_bHLH_transcription_factor_1 | NM_000474 | Tumor_marker,stemness |
| TYROBP | TYROBP_350455 | TYRO_protein_tyrosine_kinase_binding_protein | NM_198125 | Lymphocyte_infiltrate |
| VCAM1 | VCAM1_829933 | vascular_cell_adhesion_molecule_1 | NM_001078 | Leukocyte_migration |
| VEGFA | VEGFA_17121817 | vascular_endothelial_growth_factor_A | NM_001171623 | Chemokine_signaling |
| VTCN1 | VTCN1_49157 | V-set_domain_containing_T_cell_activation_inhibitor_1 | NM_024626 | Checkpoint_pathway |
| XAGE1B | XAGE1B_469547 | X_antigen_family_member_1B | NM_001097594 | Tumor_antigen |
| ZAP70 | ZAP70_673781 | zeta_chain_of_T_cell_receptor_associated_protein_kinase_70kDa | NM_001079 | T_cell_receptor_signaling |
| ZBTB46 | ZBTB46_13361440 | zinc_finger_and_BTB_domain_containing_46 | NM_025224 | Dendridic_cell |
| ZEB1 | ZEB1_74175 | zinc_finger_E-box_binding_homeobox_1 | NM_001174093 | Tumor_marker,stemness |
